# Electrocatalytic Oxygen Reduction to Produce Hydrogen Peroxide: Rational Design from Single-Atom Catalysts to Devices

**DOI:** 10.1007/s41918-022-00163-5

**Published:** 2022-09-02

**Authors:** Yueyu Tong, Liqun Wang, Feng Hou, Shi Xue Dou, Ji Liang

**Affiliations:** 1 Key Laboratory for Advanced Ceramics and Machining Technology of Ministry of Education, School of Materials Science and Engineering, Tianjin University, Tianjin, China; 2 Institute for Superconducting and Electronic Materials, Australian Institute of Innovative Materials, University of Wollongong, Innovation Campus, Squires Way, North Wollongong, NSW 2500 Australia; 3 Applied Physics Department, College of Physics and Materials Science, Tianjin Normal University, Tianjin, China

**Keywords:** Single-atom catalyst design, Electrocatalytic H_2_O_2_ production, Oxygen reduction reaction, Two-electron process

## Abstract

Electrocatalytic production of hydrogen peroxide (H_2_O_2_) via the 2e^−^ transfer route of the oxygen reduction reaction (ORR) offers a promising alternative to the energy-intensive anthraquinone process, which dominates current industrial-scale production of H_2_O_2_. The availability of cost-effective electrocatalysts exhibiting high activity, selectivity, and stability is imperative for the practical deployment of this process. Single-atom catalysts (SACs) featuring the characteristics of both homogeneous and heterogeneous catalysts are particularly well suited for H_2_O_2_ synthesis and thus, have been intensively investigated in the last few years. Herein, we present an in-depth review of the current trends for designing SACs for H_2_O_2_ production via the 2e^−^ ORR route. We start from the electronic and geometric structures of SACs. Then, strategies for regulating these isolated metal sites and their coordination environments are presented in detail, since these fundamentally determine electrocatalytic performance. Subsequently, correlations between electronic structures and electrocatalytic performance of the materials are discussed. Furthermore, the factors that potentially impact the performance of SACs in H_2_O_2_ production are summarized. Finally, the challenges and opportunities for rational design of more targeted H_2_O_2_-producing SACs are highlighted. We hope this review will present the latest developments in this area and shed light on the design of advanced materials for electrochemical energy conversion.

**Graphical abstract:**

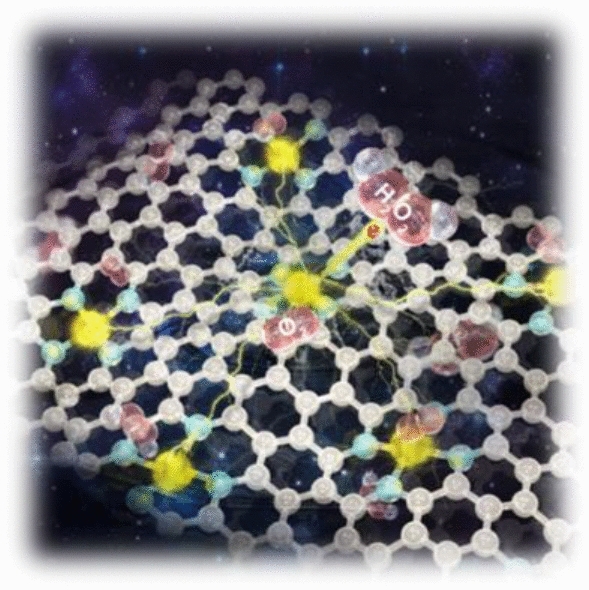

## Introduction

Hydrogen peroxide (H_2_O_2_) is an ecofriendly oxidant with high oxidation potentials over the entire pH range (e.g., pH = 0, *E*_0_ = 1.763 V vs. standard hydrogen electrode (SHE); pH = 14, *E*_14_ = 0.878 V vs. SHE), giving it outstanding bleaching and antiseptic capabilities that lead to many applications in the pulp and paper, textiles, healthcare, and wastewater treatment industries [1, 2]. As one of the 100 most important chemicals in the world, H_2_O_2_ has attracted unprecedented attention amid the coronavirus-19 (COVID-19) pandemic that started in late 2019 [3]. During the COVID-19 crisis, the sanitization capacity of H_2_O_2_ has been further applied for various personal hygiene needs, such as in first-aid kits for minor cuts, antiseptic treatments, and mouthwashes for removing mucus and other mouth irritants. Thus, demands from both the industrial and medical communities have stimulated increases in the production of H_2_O_2_. It is predicted that H_2_O_2_ production will increase to 1.2 million tons by 2027, which represents a 4% annual growth rate [4–6].

The large-scale anthraquinone process constitutes up to 95% of global H_2_O_2_ production, but at the cost of high levels of energy consumption and waste emission [1, 4]. Typically, in this process, anthraquinone is first hydrogenated by H_2_ with a Pd-based catalyst and sequentially oxidized by O_2_ in alkaline organic solvents, and then, it is subjected to a series of complex distillation and impurity separation steps to recover highly concentrated H_2_O_2_ [~ 70 wt.% (wt.% means the weight fraction)] from byproducts and anti-decomposition stabilizers of H_2_O_2_ [1, 7, 8]. In addition to the high cost of Pd-based catalysts, risks in the centralized transport, storage, and handling of concentrated H_2_O_2_ are also significant after production and before shipment to end-users. Thus, this process is only economically feasible for large-scale production and consumption of concentrated H_2_O_2_ (> 40 × 10^3^ t per annum) [9].

However, in other scenarios, such as in the medical disinfection and cosmetic industries, diluted H_2_O_2_ ($\leqslant$ 3 wt.%) rather than concentrated H_2_O_2_ is actually required [10, 11]. Especially for sanitation and disinfection in backward and remote regions, cost-effective and on-site production of H_2_O_2_ (i.e., decentralized technologies) is more commercially viable than transporting H_2_O_2_ over long distances. In this regard, the electrochemical synthesis of H_2_O_2_, including either direct combination of H_2_/O_2_ or reduction in O_2_ through ORR, can be promising alternatives to the anthraquinone process. In addition to producing H_2_O_2_, these processes can also be integrated with fuel-cell systems, thereby generating electric power while continually producing H_2_O_2_ on-site.

For direct H_2_O_2_ synthesis from H_2_/O_2_, diluted H_2_ and O_2_ gases stored in high-pressure diluents (e.g., carbon dioxide, nitrogen, argon) are bubbled into the anodic and cathodic chambers of the H_2_/O_2_ fuel cell, respectively [12]. With the help of Pd-based catalysts (e.g., Pd–Sn and Pd–Au alloys), H_2_O_2_ can be produced [13, 14]. Although this route enables the on-site production of dilute H_2_O_2_, the unavoidable risks of the flammable/explosive feed gases and the comparatively low selectivity for H_2_O_2_ production intrinsically impede its viability for practical applications [15, 16]. Despite the fact that some additives (e.g., acid promoters and halide ions) have been used to boost the selectivity for H_2_O_2_ in this method, the accompanying issue of subsequent H_2_O_2_ purification significantly increases the cost [17, 18].

**Fig. 1:**
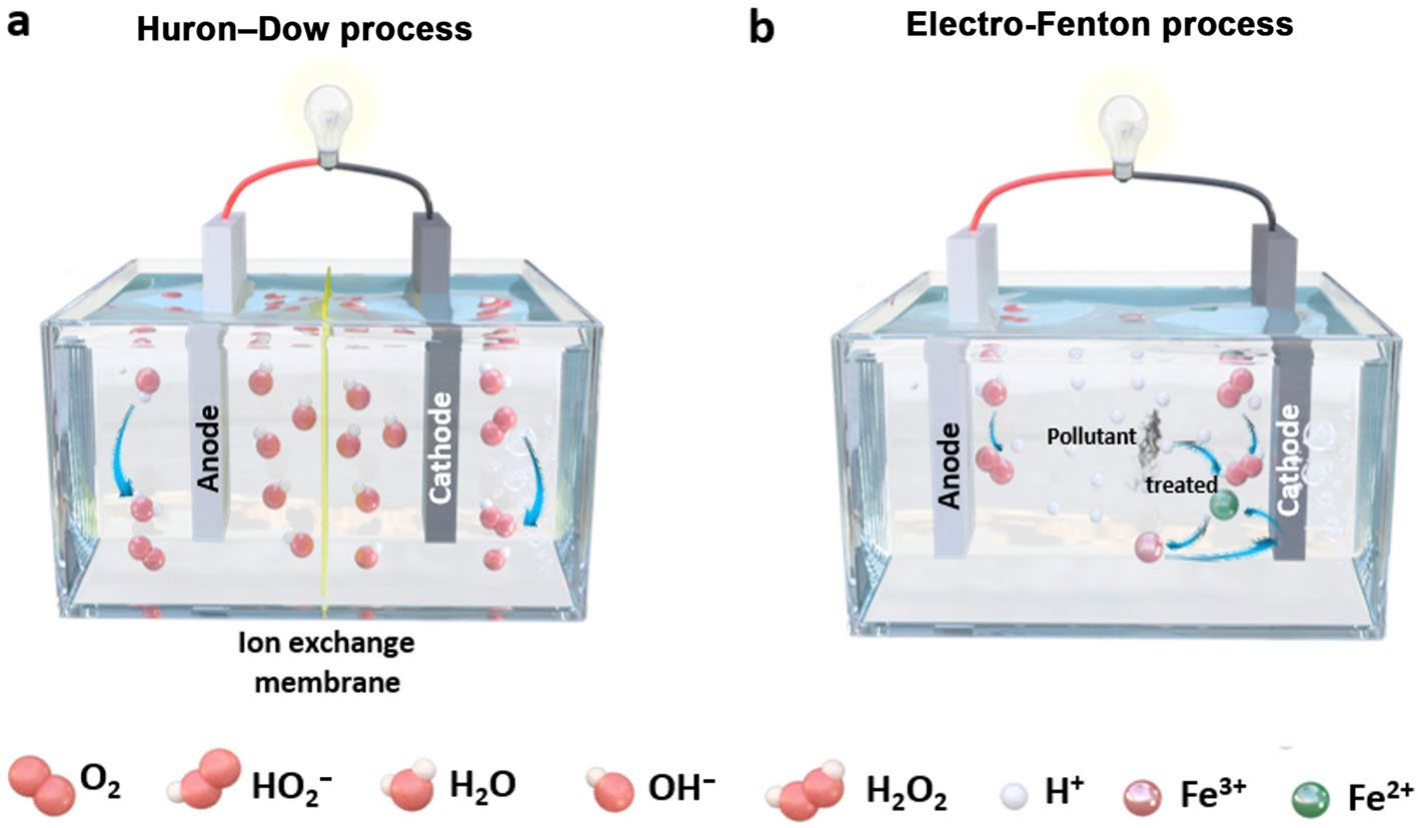
Schematic illustrations of various electrochemical 2e^−^ ORR processes for H_2_O_2_ production: **a** Huron–Dow process, **b** Electro-Fenton process

In comparison, the 2e^−^ ORR route offers a simple and low-risk method for continuous and in situ production of high-value-added H_2_O_2_ under relatively mild conditions in a decentralized way. Electrochemical 2e^−^ ORR was first commercialized in the pulp and paper bleaching process in 1991 and is known as the Huron–Dow process for on-site production of dilute H_2_O_2_ in an alkaline electrolyte (Fig. 1a) [7]. However, the high alkalinity of the electrolyte requires immediate consumption of the produced H_2_O_2_; otherwise, the alkaline H_2_O_2_ will decompose very quickly [19, 20]. As a modification of the Huron–Dow process, the electro-Fenton process takes place in a sodium sulfate solution at pH = 3, in which H_2_O_2_ can be continuously produced at the cathode at concentrations ranging from 10 μg g^−1^ to 2 wt.% (Fig. 1b) [21]. The extra Fe^2+^ and H_2_O_2_ produced generate hydroxyl radicals (·OH) in situ for degradation of organic pollutants, while Fe^2+^ is recovered on the cathode [22].

To achieve efficient H_2_O_2_ production, catalysts with both high activity and selectivity for the electrocatalytic 2e^−^ ORR process are a prerequisite. A diverse variety of electrocatalysts, including carbon-based materials, transition metal alloys, and others, have been developed and utilized for H_2_O_2_ production via the 2e^−^ ORR route [23–29]. Electrocatalysts, which are capable of enhancing H_2_O_2_ synthesis by lowering the kinetic barriers of the 2e^−^ ORR and suppressing possible side reactions, are considered essential for achieving the ultimate commercialization of this technology [30]. Over the past years, a variety of catalysts for H_2_O_2_ production through the 2e^–^ ORR process have been developed, including homogeneous molecular catalysts and heterogeneous catalysts. For example, cobalt macrocycles [31–33], molecular manganese compounds [34, 35], and copper complex catalysts [36, 37] have been demonstrated to be promising homogeneous molecular catalysts. Heterogeneous catalysts include pure carbon materials (i.e., hierarchical porous carbon materials [38] and defect-rich carbon nanotube (CNT) catalysts [39]), heteroatom-modified carbon materials (i.e., N-doped carbon materials [40, 41], O-doped CNTs [42], O-doped graphene [43], N,F-codoped carbon nanocages [44], and N,S-codoped mesoporous carbon materials [45]), transition metal-based materials (i.e., Co particles loaded on carbon supports, partially pyrolyzed Ni–Fe binary metal–organic framework catalysts [46]), and single-atom catalysts (SACs) with various metal sites and supports, many of which show good selectivity and activity.

In particular, SACs with isolated, well-defined, and coordinated metal atoms dispersed on supports have been investigated for various reactions, and they show promising electrocatalytic activity and selectivity [3, 47–57]. Nearly 100% of isolated metal atoms, which are covalently coordinated or ionic, interact with nearby atoms supported on the surface and efficiently participate in electrocatalytic reactions, enabling the highly efficient and inexpensive utilization of noble metals and enhanced mass activity [30, 58]. Compared with conventional supported nanoparticle catalysts, the very uniform distribution of active metal centers with SACs also makes them a simple and ideal theoretical model for investigating intrinsic structure-performance correlations and understanding their electrocatalytic mechanisms on the atomic scale [59, 60]. Endowed with these properties, SACs provide an ideal shortcut for achieving high-efficiency and electrocatalytic H_2_O_2_ production. To date, a number of strategies based on isolating individual metal atoms via control of catalyst loading or metal alloying have been reported for the fabrication of catalysts for H_2_O_2_ production [8, 61]. Considering these findings, we believe that it is not only necessary but also urgent to present a comprehensive summary of strategies for atomic screening and coordination modulation of high-performance SACs for electrocatalytic H_2_O_2_ production through the 2e^−^ ORR process.

Herein, we will elaborate the fundamental mechanisms of direct H_2_O_2_ production by the 2e^−^ ORR and focus on strategies for the rational design of single-atom electrocatalysts at the atomic scale to achieve high activity, selectivity, and stability. The influence of metal atom centers and coordination of neighboring atoms on electrocatalytic behavior are discussed in detail. Additionally, advanced cell design strategies for achieving enhanced electrocatalytic performance are also presented. Knowing the theoretical and experimental aspects of these controlling factors will make future development of single-atom catalysts more performance-oriented. Furthermore, recent advances in designing single-atom electrocatalysts for H_2_O_2_ production through the 2e^−^ ORR are illustrated in detail. Finally, major challenges and perspectives for efficient design of electrocatalysts and reaction conditions facilitating transfer from lab-scale to plant-size applications are summarized.

## Mechanism of H_2_O_2_ Production from ORR

**Fig. 2:**
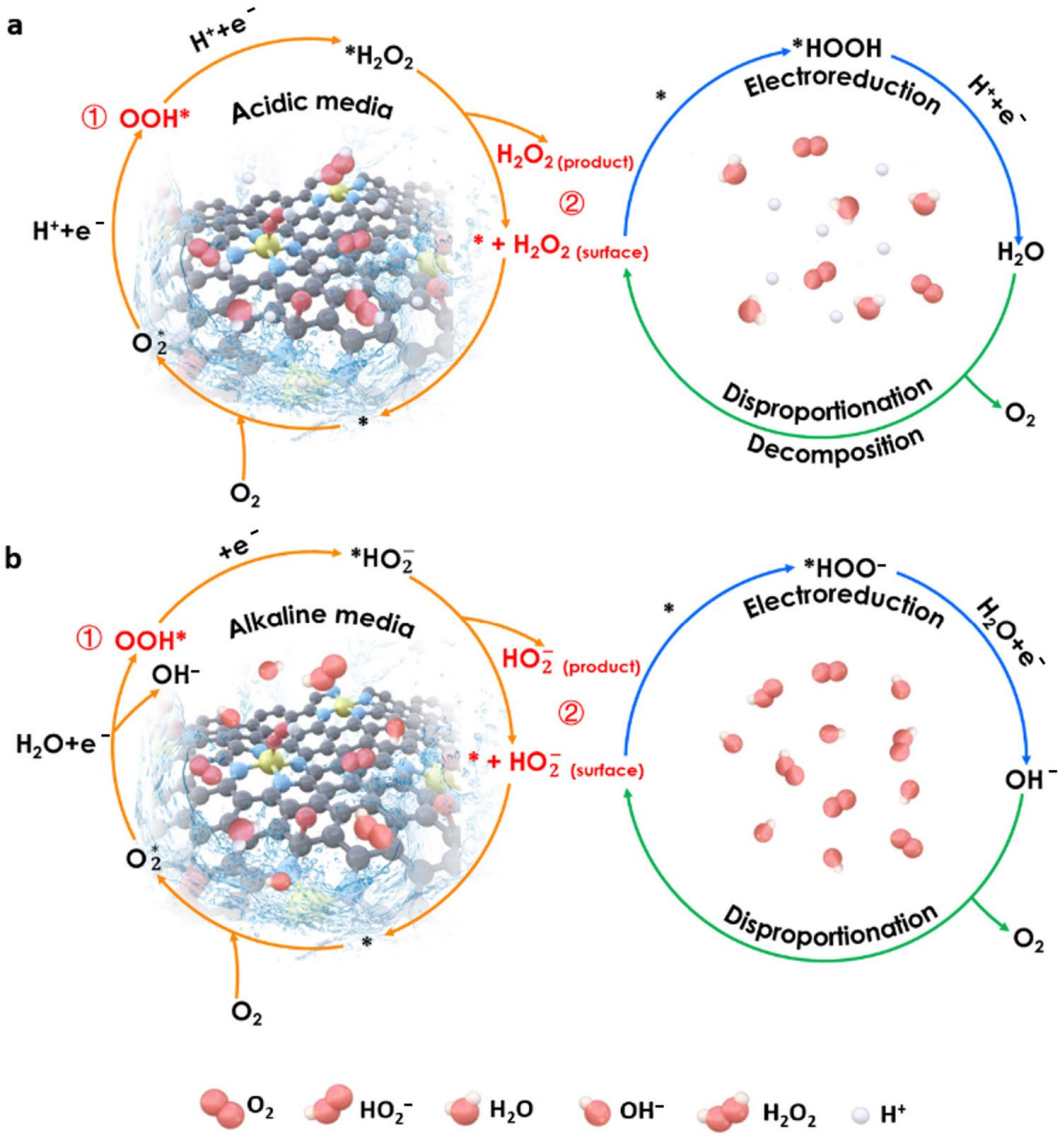
Schematic illustrations of the 2e^−^ ORR pathway for H_2_O_2_ production in **a** acidic and **b** alkaline media, where the asterisk “*” represents the active sites of catalysts

The electrochemical ORR involves multielectron transfer processes, either generating the target H_2_O_2_ by the 2e^−^ pathway or competitively producing H_2_O through a 4e^−^ route. The 4e^−^ route for H_2_O production (acidic conditions: O_2_ + 4H^+^  + 4e^−^  → 2H_2_O; or alkaline conditions: O_2_ + 2H_2_O + 4e^−^  → 4OH^−^) has long been studied for application in metal-air batteries and fuel cells, which is not within the scope of this review [7, 62–65]. The mechanism of the electrochemical ORR proceeding via the 2e^−^ pathway is depicted in Fig. 2 and described as follows [49, 66, 67]:

In acidic media,

1.1a $$* + {\text{ O}}_{{2}} + {\text{ H}}^{ + } + {\text{ e}}^{ - } \to \, ^*{\text{OOH}}$$

1.2a $$^*{\text{OOH}} + {\text{H}}^{ + } + {\text{ e}}^{ - } \to {\text{ H}}_{{2}} {\text{O}}_{{2}} + *$$

2a $${\text{O}}_{{2}} + {\text{ 2H}}^{ + } + {\text{ 2e}}^{ - } \to {\text{ H}}_{{2}}{\text{O}}_{{2}}\ \left( {{\text{pH < 11}}.{6}} \right)$$

3.1a $${\text{H}}_{{2}} {\text{O}}_{{2}} + {\text{ 2H}}^{ + } + {\text{ 2e}}^{ - } \to {\text{ 2H}}_{{2}} {\text{O}}$$

3.2a $${\text{2H}}_{{2}} {\text{O}}_{{2}} \to {\text{ 2H}}_{{2}} {\text{O }} + {\text{ O}}_{{2}}$$

In alkaline media,

1.1b $$* + {\text{ O}}_{{2}} + {\text{ H}}_{{2}} {\text{O }} + {\text{ e}}^{ - } \to \, ^*{\text{OOH}} + {\text{OH}}^{ - }$$

1.2b $${\text{OOH}}^* + {\text{ e}}^{ - } \to {\text{HO}}_{{2}}^{ - } + *$$

2b $${\text{O}}_{{2}} + {\text{ H}}_{{2}} {\text{O }} + {\text{ 2e}}^{ - } \to {\text{ HO}}_{{2}}^{ - } + {\text{OH}}^{ - } \,\left( {{\text{pH}} > {11}.{6}} \right)$$

3.1b $${\text{HO}}_{{2}}^{ - } + {\text{ H}}_{{2}} {\text{O }} + {\text{ 2e}}^{ - } \to {\text{ 3OH}}^{ - }$$

3.2b $${\text{2HO}}_{{2}}^{ - } \to {\text{ 2OH}}^{ - } + {\text{O}}_{{2}}$$

The asterisk “*” represents the active sites of catalysts in the above equations.

For these processes, it is worth mentioning that *OOH is generated in both the 2e^−^ and 4e^−^ ORR routes, and the peroxide species may be further reduced to H_2_O (Eq. 3.1a) or OH^−^ (Eq. 3.1b) via another 2e^−^ pathway or may disproportionate to O_2_ and H_2_O (Eq. 3.2a) or O_2_ and OH^−^ (Eq. 3.2b) [68, 69]. Consequently, the generation of H_2_O_2_ is fundamentally determined by two factors: the binding strength of the *OOH intermediate and the facility of desorption and the stability of the peroxide species (H_2_O_2_ or HO_2_^−^) produced on the active sites of the catalyst. Thus, after the adsorption of O_2_ molecules on catalytic active sites, catalysts that exhibit moderately strong binding with the *OOH intermediate tend to favor high selectivity for H_2_O_2_ production. In contrast, catalysts that form excessively strong bonds with the *OOH species easily split the O–O bond to produce undesired H_2_O, while those that form an overly weak bond may be unable to activate the absorbed O_2_ to form *OOH [60]. Minimizing product accumulation near the electrode and rapidly releasing active sites to facilitate mass transfer in the cell are equally important for the design of electrocatalysts that allow fast detachment of product peroxide species [7]. Further optimization of electrocatalytic conditions, such as electrolyte type, oxygen input, temperature, and cell configuration, can also affect the desorption and stability of peroxide products, which will also be discussed in this review.

## Design of H_2_O_2_-Producing Electrocatalysts

### Theoretical Design of H_2_O_2_-Producing Electrocatalysts

**Fig. 3:**
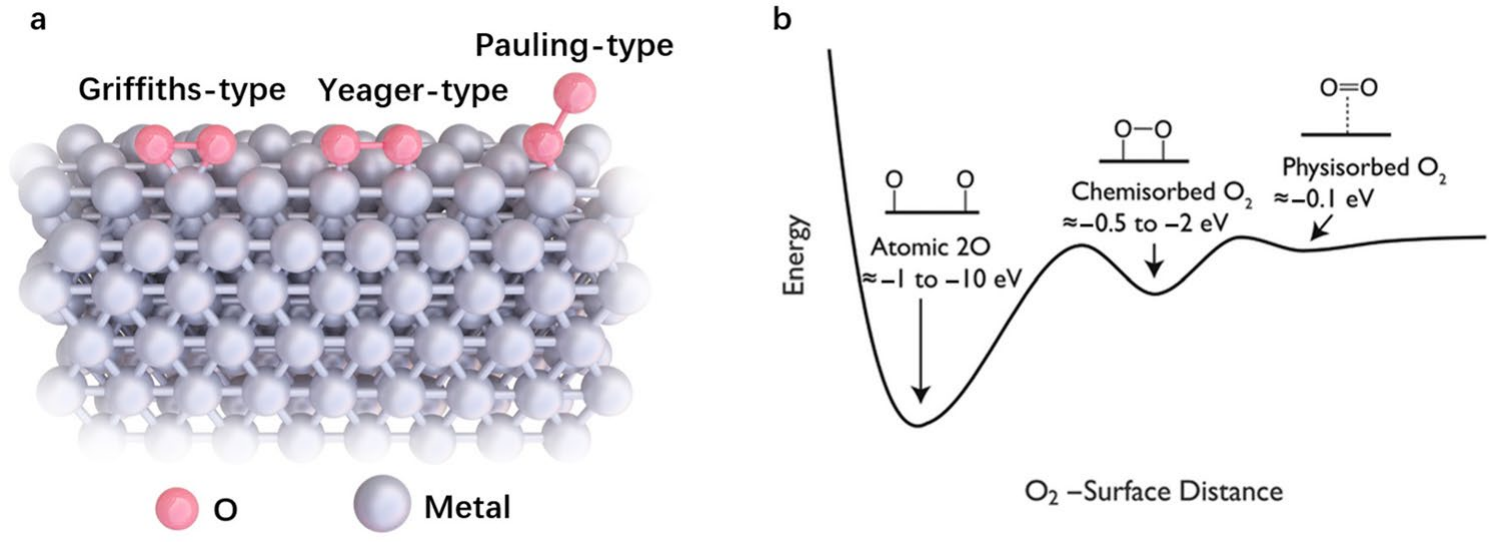
**a** Schematic illustration of the different structures of O_2_ adsorbed on metal active sites. **b** Potential energy diagram of O_2_ adsorbed on a metal active site. Reprinted with permission from Ref. [72]. Copyright © 2018, American Chemical Society

Theoretically, ideal 2e^−^ ORR electrocatalysts for H_2_O_2_ synthesis should have low activation barriers for forming *OOH to achieve high activity and a high dissociation barrier for *OOH to achieve high selectivity [70]. Nonetheless, appropriate adsorption of O_2_ on active sites is a prerequisite. Metal active sites, such as Pt, usually have strong interactions with O_2_ and exhibit two structure types for O_2_ adsorption (Fig. 3a): the Griffiths-type (i.e., side-on adsorption of an O_2_ molecule on one metal atom) and the Yeager-type (i.e., side-on bridging adsorption of an O_2_ molecule on 2 adjacent metal atoms) [71]. They lead to chemisorbed O_2_, as shown in Fig. 3b, with adsorption energy ranging from − 0.5 to − 2 eV. In this case, O_2_ can be directly reduced to H_2_O through the 4e^−^ pathway. In contrast, end-on adsorption (Pauling-type) is more desirable for generating H_2_O_2_. Pauling-type adsorption, with an adsorption energy less than − 0.1 eV, can be categorized as physisorption or weak chemisorption, which is not thermodynamically favorable for the side-on configuration. For SACs, the intrinsically isolated metal sites on the surface are dispersed on the atomic scale, resulting in a high preference for end-on O_2_ adsorption over side-on adsorption [31, 72]. Therefore, the chance of O–O bond breaking is greatly reduced on SACs, which indicates that SACs have intrinsic advantages for H_2_O_2_ production by the 2e^−^ ORR.

**Fig. 4:**
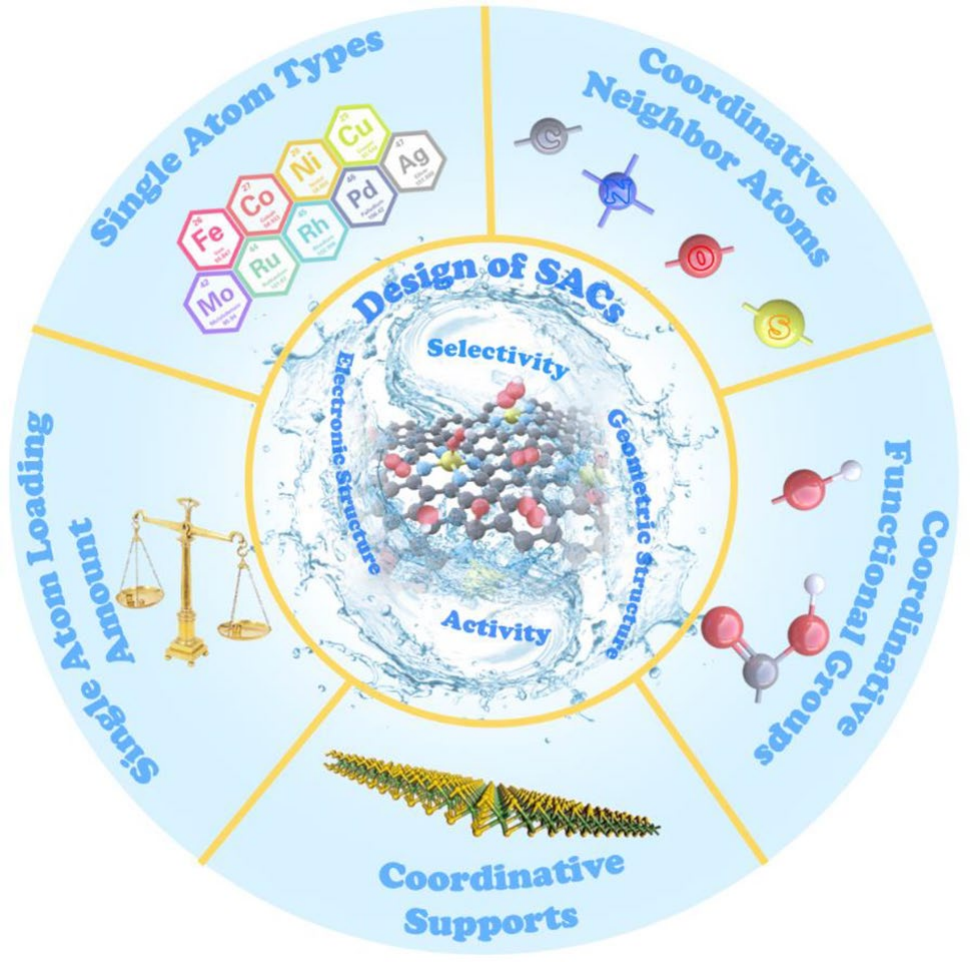
Schematic illustration of modification strategies for single-atom sites and coordination environments for SACs and correlations with electronic structure, geometric structure, and electrocatalytic performance

Along with adsorption of O_2_, fine modification of metal atom centers and careful tuning of their coordination environments (i.e., neighboring atoms and the coordination numbers) are two important factors in the design of SACs that determine the electronic and geometric effects of catalysts, as shown in Fig. 4. Electronic effects mainly control the activity and partially modify the selectivity of a catalyst, while geometric effects largely affect selectivity [7].

**Fig. 5:**
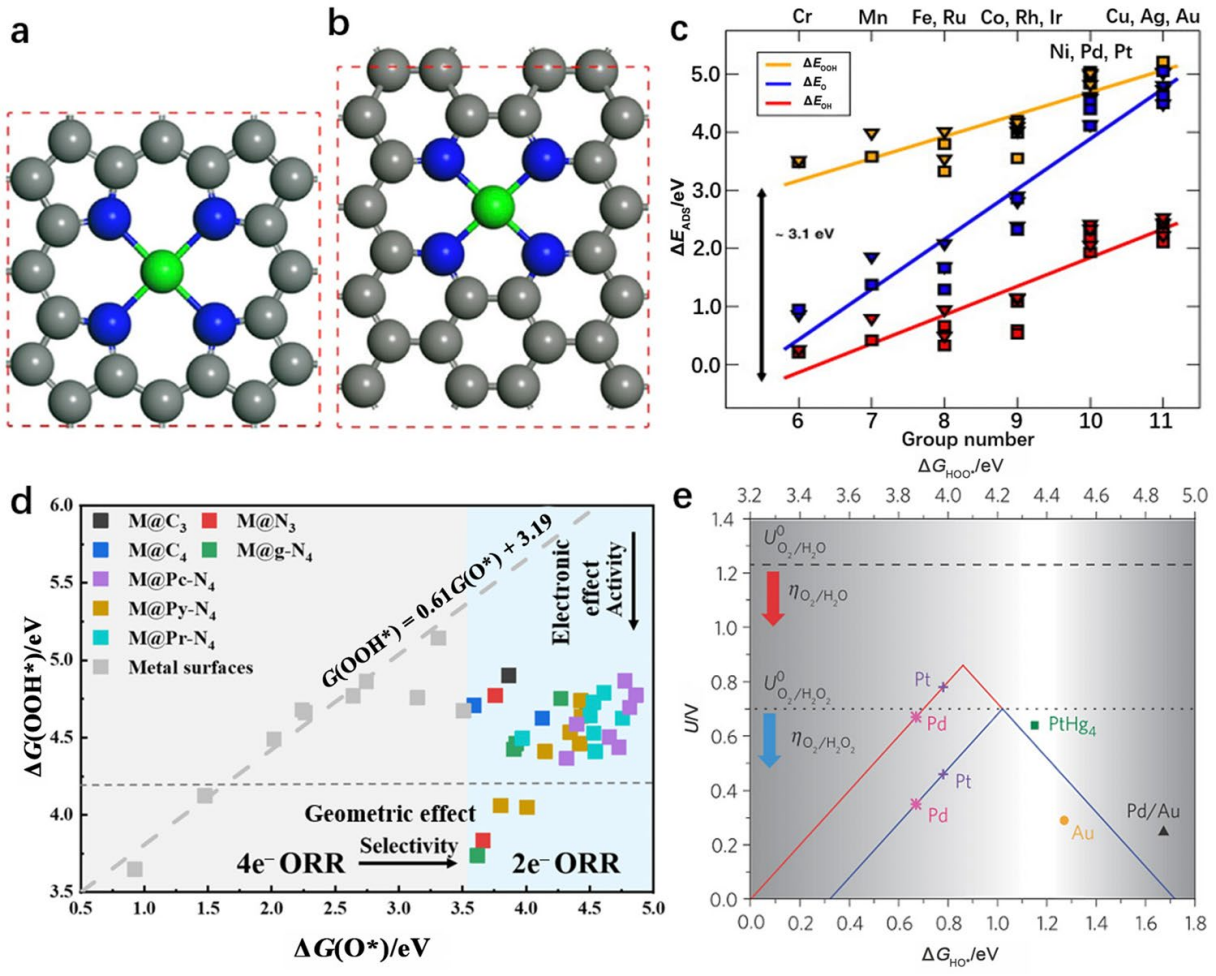
**a** Isolated metal center anchored with four pyrrole nitrogen atoms (pyrrole–N_4_) in SACs. **b** Isolated metal center anchored with four pyridine nitrogen atoms (pyridine–N_4_) in SACs. The green, blue, and gray colors represent transition metal (TM), N, and C atoms, respectively. Unit cells are outlined in red. Reprinted with permission from Ref. [74]. Copyright © 2018, Springer Nature. **c** Trends in binding energy of ORR intermediates for M–pyrrolic–N_4_ (triangles) and M–pyridine–N_4_ (squares)-functionalized graphitic materials. Reprinted with permission from Ref. [75]. Copyright © 2013, Elsevier. **d** Variations in Δ*G*(O*) and Δ*G*(*OOH) for 31 studied TM SACs with various coordination environments. Reprinted with permission from Ref. [76]. Copyright © 2019, American Chemical Society. **e** Theoretically calculated ORR volcano plot for the 2e^−^ (blue) and 4e^−^ (red) pathways with the binding energy of *OH or *OOH as a descriptor (Δ*G*_HO*_ or Δ*G*_HOO*_, respectively). Reprinted with permission from Ref. [77]. Copyright © 2013, Springer Nature

In addition, a series of theoretical simulations have been conducted from the perspectives of metal active site screening and coordination environment modification to explore appropriate SACs for H_2_O_2_ production through the 2e^−^ electrocatalytic ORR process. Recently, ORR processes occurring at various transition metal single-atom sites anchored by four pyrrolic nitrogen atoms (M–pyrrolic–N_4_, Fig. 5a) and four pyridine nitrogen atoms (M–pyridine–N_4_, Fig. 5b) on the carbon network have been investigated by density functional theory (DFT), as shown in Fig. 5c [73]. With increasing group number (the horizontal axis of Fig. 5c), the energy needed to adsorb the oxygen-containing intermediates gradually increased for M–N_4_ active sites, resulting in a correspondingly weakened binding strength. In terms of metal centers, Co, Rh, Ir, Ni, Pd, and Pt single-atom sites were predicted to be promising candidates for electrocatalyzing the 2e^−^ ORR for H_2_O_2_ production due to their relatively moderate energy for adsorption of oxygen intermediates (i.e., *OOH, *O, and *OH).

Nevertheless, the actual electrocatalytic activity and selectivity of SACs are also heavily dependent on the local geometries of the single-atom centers. Considering this, Guo et al. performed DFT calculations to compare the electrocatalytic activity and selectivity for H_2_O_2_ production with 31 transition metal SACs exhibiting various coordination configurations [76]. They further combined multiple-variable analysis with machine learning to determine structure-performance correlation of SACs for H_2_O_2_ production, considering both electronic and geometric effects. In this study, the coordination configurations, including those of graphene (M@C_3_, M@C_4_), N-doped graphene (M@g–N_4_), boron nitride monolayer (M@N_3_), phthalocyanine–N_4_ (M@Pc–N_4_), pyrphyrin–N_4_ (M@Py–N_4_), and porphyrin–N_4_ (M@Pr–N_4_), were investigated (Fig. 5d). Compared with the corresponding metal surfaces, these SACs presented weaker binding to the O* species and were more appropriate for H_2_O_2_ production. When the adsorption energy of O* (Δ*G*_O*_) is greater than 3.52 eV, the adsorption of *OOH is improved for SACs.

In this study, it was also found that after an O_2_ molecule reacted with an H^+^/e^−^ pair, the coordination support served as a donor of electrons to *OOH, which is conducive to stronger *OOH adsorption. As a result, the M@Pc–N_4_, M@Py–N_4_, and M@Pr–N_4_ configurations exhibited completely different catalytic properties even with the same metal center. Thus, the coordination environments can be tuned to precisely control the adsorption capability of the metal center and achieve the desired electrocatalytic properties. In particular, Ag, Au, and Pd normally have weak affinities for oxygen, which leads to a significantly reduced bond hybridization between metal and oxygen, which guarantees improved selectivity but relatively lower activity in H_2_O_2_ synthesis. A single Zn atom centered within a phthalocyanine ligand (Zn@Pc–N_4_) showed moderate charge transfer for *OOH adsorption; it was comparable with that of PtHg_4_, which was predicted to be the most suitable electrocatalyst for the 2e^−^ ORR producing H_2_O_2_.

Unlike anchoring single atoms on carbonaceous supports, loading them onto a less reactive host metal generates single-atom alloy (SAA) catalysts. As shown in Fig. 5e, various SAA catalysts were computationally screened for H_2_O_2_ production [77]. In the right-hand branch of the 2e^−^ volcano, the reduction in O_2_ to *OOH controls H_2_O_2_ production and is the rate-limiting process because a high energy is required to absorb *OOH (∆G_*OOH_, weak binding). At the same time, H_2_O_2_ production over the materials shown in the left-hand branch is limited by the reduction in *OOH to H_2_O_2_ due to low values for ∆G_*OOH_ (strong binding). Catalysts situated on the peak of the 2e^−^ volcano should be those most active for electrocatalytic H_2_O_2_ production. Considering this, alloying Pt with Hg to form PtHg_4_ would effectively weaken the originally strong binding between Pt and the *OOH intermediate, resulting in a *OOH binding energy close to that at the volcano peak, i.e., neither too strong nor too weak. Compared to Pt, PtHg_4_ should exhibit greatly enhanced reactivity in the 2e^−^ ORR producing H_2_O_2_, and an extremely high mass activity of over 25 A g^−1^ could be expected for the PtHg_4_ electrocatalyst. Thus, theoretical screening of SACs with proper metal sites and coordination environments enables researchers to conduct experiments in a more rational and directed way.

### Strategies for Designing SACs for Electrocatalytic H_2_O_2_ Production

To boost the capacities of SACs, the choices of the isolated metal and the coordination environment are both essential. As shown by numerous previous investigations, modulating isolated transition-metal centers and their coordination sphere is a highly efficient method for tuning the electrocatalytic activity, selectivity, and stability of SACs. Consequently, the performance of SACs is highly dependent on the type, coordination sphere, and steric environment of the active metal centers and the corresponding supports [78]. In this section, we will discuss recent advances in the modification of single atoms and their coordination conditions in producing SACs for electrocatalytic H_2_O_2_ production.

#### Modification of Single-Atom Metal Centers

Currently, the use of noble metal catalysts in the 2e^−^–ORR for H_2_O_2_ synthesis is attracting increasing interest. As a representative material, Pd-based catalysts have already been widely used in the industrial anthraquinone process. Although some noble metal-based catalysts (i.e., Pt nanoparticles) are well known to catalyze the 4e^−^ ORR process with high intrinsic activity, tuning their ORR to the 2e^−^ pathway by isolating active metal centers is considered a highly efficient approach for H_2_O_2_ production.

**Fig. 6:**
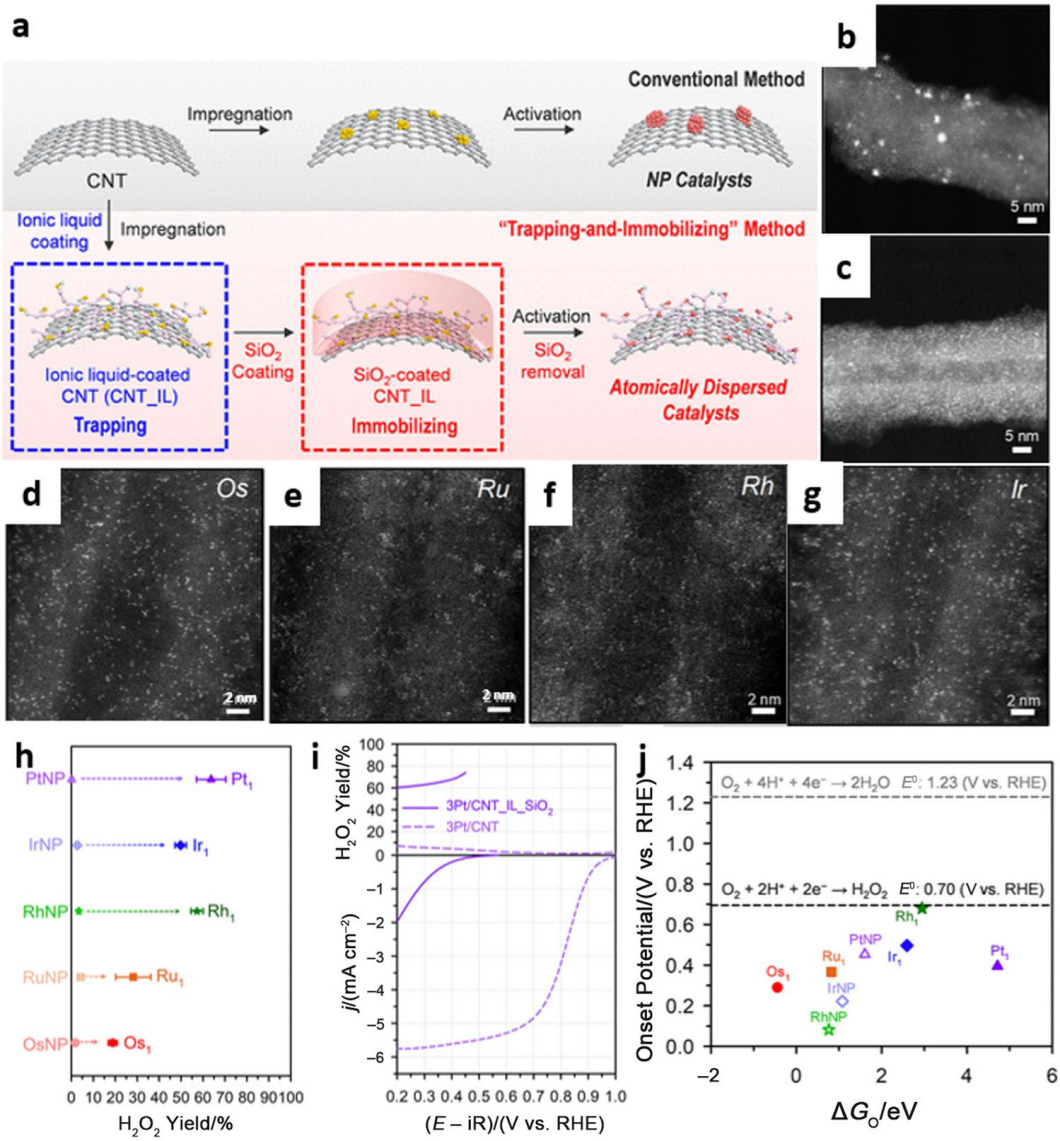
**a** Schematic for three types of catalysts: Pt/CNT, Pt/CNT_IL, and Pt/CNT_IL_SiO_2_. **b** High-angle annular dark-field scanning transmission electron microscopy (HAADF-STEM) image of the 3Pt/CNT catalyst. HAADF-STEM images of **c** 3Pt/CNT_IL_SiO_2_ catalyst, **d** 3Os/CNT_IL_SiO_2_, **e** 1.5Ru/CNT_IL_SiO_2_, **f** 1.5Rh/CNT_IL_SiO_2_ and **g** 3Ir/CNT_IL_SiO_2_. **h** H_2_O_2_ production with M/CNT (denoted as MNP) and M/CNT_IL_SiO_2_ (denoted as M_1_) catalysts at − 0.5 mA cm^–2^. **i** ORR polarization curves and H_2_O_2_ yields for 3Pt/CNT_IL_SiO_2_. **j** Volcano-shaped relationship between O binding energy (Δ*G*_O_) and onset potentials for the 2e^–^ ORR pathway with M_1_ and MNP catalysts. Reprinted with permission from Ref. [80]. Copyright © 2020, American Chemical Society

The primary challenge in preparing noble metal-based SACs is to stabilize the highly dispersed single atoms during the 2e^−^–ORR process [79]. To achieve this, a SiO_2_ layer was adopted to immobilize the single noble metal atoms during thermal activation, which were first trapped in ionic liquid (IL)-derived Os, Ru, Rh, Ir, and Pt-doped carbonaceous layers and then coated on carbon nanotubes (CNTs), as shown in Fig. 6a–g [80]. The resulting family of SACs (*x*M/CNT_IL_SiO_2_, for which M is Os, Ru, Rh, Ir, or Pt, and *x* is the doping amount in wt.%) that were synthesized by this “trapping-and-immobilizing” method all showed higher selectivity for H_2_O_2_ production than their nanoparticle counterparts (Fig. 6h). It was found that the choice of metal center further regulated the electrocatalytic properties of these SACs for H_2_O_2_ synthesis, with 3Pt/CNT_IL_SiO_2_ showing the highest selectivity of 63.7% (Fig. 6i) and 1.5Rh/CNT_IL_SiO_2_ exhibiting the highest activity (Fig. 6j). DFT calculations further showed that weak binding of the *OOH species is desirable for achieving high selectivity, but a moderate binding energy is necessary for achieving high activity; this requires researchers to consider the trade-off between activity and selectivity during the design of catalysts.

**Fig. 7:**
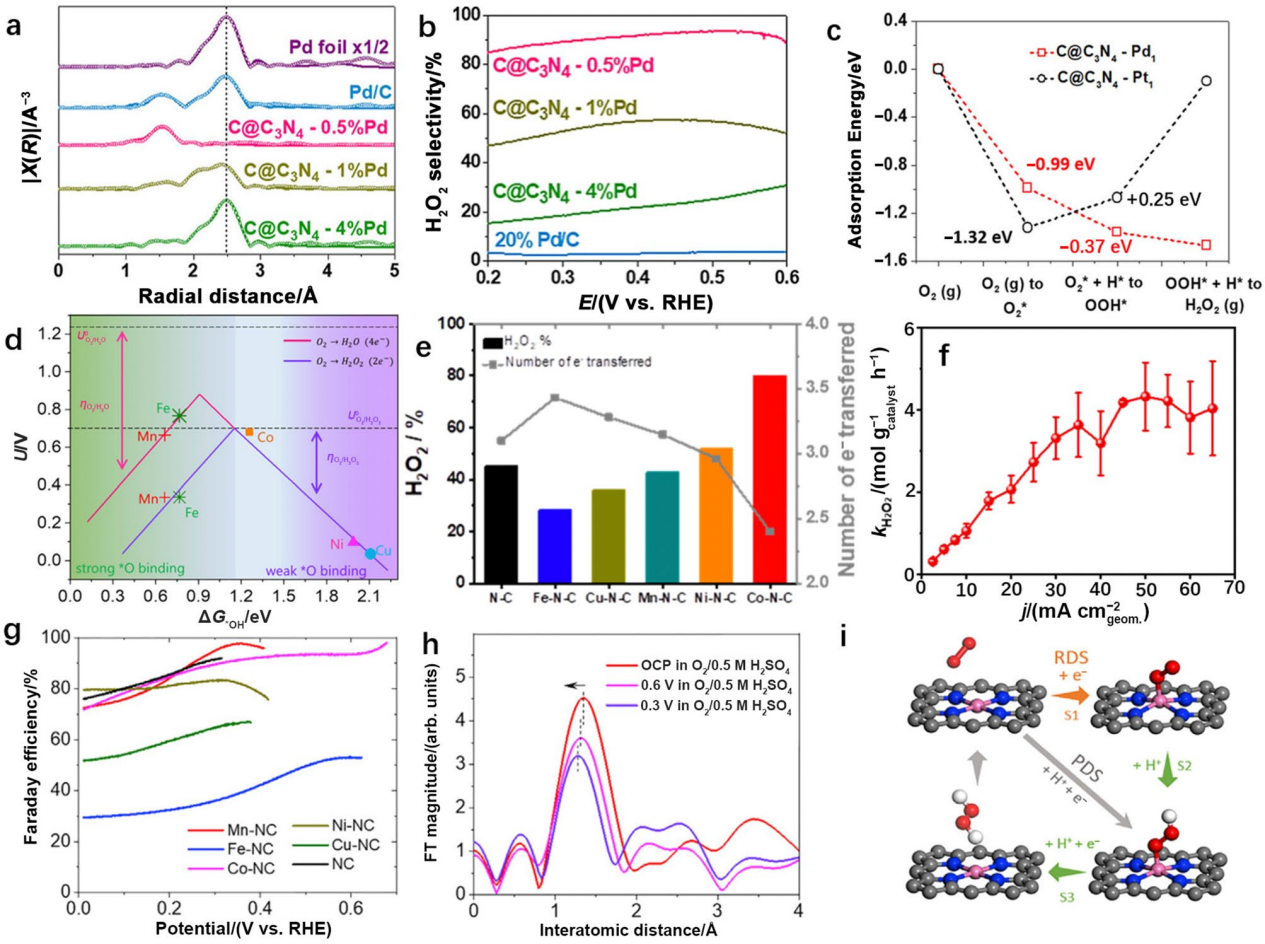
**a** X-ray absorption near edge structure (XANES) spectra for the Pd K edge. The symbols indicate experimental data, and the solid lines indicate fitted results. **b** H_2_O_2_ selectivity of Pd catalysts via 2e^−^ ORR measured in O_2_-saturated 0.1 M HClO_4_ solution (1 M = 1 mol L^−1^). **c** Energy profile of intermediates adsorbed on atomic Pd and Pt during the ORR at *U* = 0 V. Panels (**a–c**) are reprinted with permission from Ref. [81]. Copyright © 2019, John Wiley and Sons. **d** Volcano plot of the ORR via the 2e^−^ or 4e^−^ route for M SACs (M = Mn, Fe, Co, Ni, and Cu). **e** H_2_O_2_ selectivity and the number of electrons transferred with M SACs (M = Mn, Fe, Co, Ni, and Cu) at 0.1 V vs. RHE in 0.5 M H_2_SO_4_. **f** H_2_O_2_ production rate measured in a commercial microflow cell at 50 mA cm^−2^ with a Co–N–C electrode in O_2_-saturated 0.1 M (1 M = 1 mol L^−1^) KOH. Panels (**e–f**) are reprinted with permission from Ref. [82]. Copyright © 2019, American Chemical Society. **g** Faradaic efficiency of H_2_O_2_ production in O_2_-saturated 0.1 M HClO_4_ via 2e^−^ ORR over M SACs (M = Mn, Fe, Co, Ni, and Cu). **h** In situ extended X-ray absorption fine structure (EXAFS) spectra of Co SAC during the 2e^−^ ORR ( 1 Å = 1×10^−10^ m). **i** Possible reaction mechanism of the 2e^−^ ORR over a Co SAC. Panels (**d**, **g–i**) are reprinted with permission from Ref. [83]. Copyright © 2020, Elsevier

In addition to the abovementioned precious metals, a Pd-based SAC has also been applied for electrocatalytic production of H_2_O_2_ via the 2e^−^ ORR. To synthesize this catalyst, g–C_3_N_4_ was used as a host to immobilize noble metal atoms. Specifically, carbon black-supported g–C_3_N_4_ was first impregnated in a Pd precursor solution to trap Pd atoms in g–C_3_N_4_. Then, g–C_3_N_4_ was formed at 400 °C to immobilize 0.5 wt.% single-atom Pd on carbon black (C@C_3_N_4_–0.5%Pd). It turned out that both high activity (8.5 A ${\mathrm{mg}}_{\mathrm{Pd}}^{-1}$ at 0.2 V vs. RHE) and selectivity (90% at 0.55 V vs. RHE) for H_2_O_2_ production was achieved on C@C_3_N_4_–0.5%Pd in 0.1 M HClO_4_ (Fig. 7a, b) [81]. DFT calculations revealed that C@C_3_N_4_–0.5%Pd exhibited weaker binding with oxygen than Pt (Fig. 7c), leading to enhanced activity. Simultaneously, a downhill energy diagram for producing H_2_O_2_ on C@C_3_N_4_–0.5%Pd leads to improved selectivity.

To compare the electrocatalytic performance of reported SACs in H_2_O_2_ production, the critical parameters of these materials are summarized in Table 1.

**Table 1:** Comparison of recently reported electrocatalysts for H_2_O_2_ production via the 2e^−^ ORR

Catalyst	Electrolyte	Mass activity /(A g^−1^)	Selectivity/%	Yield rate /(mol ${\mathrm{g}}_{\mathrm{cat}.}^{-1}$ h^−1^)	References
3Pt/CNT_IL_SiO_2_	0.1 M HClO_4_	N/A	63.7 (0.34 V vs. RHE)	N/A	[3]
C@C_3_N_4_–0.5%Pd	0.1 M HClO_4_	8.5 A ${\mathrm{mg}}_{\mathrm{Pd}}^{-1}$ (0.2 V vs. RHE)	90 (0.55 V vs. RHE)	N/A	[81]
Co–N–C	0.5 M H_2_SO_4_	3.37 (0.65 V vs. RHE)	80 (0.1 V vs. RHE)	4.33 (50 mA cm^−2^)	[82]
Co–N–C	0.1 M HClO_4_	11.07 (0.65 V vs. RHE)	> 90 (0.6 V vs. RHE)	0.275 (0.4 V vs. RHE)	[83]
COF–366–Co	0.1 M KOH	0.242 (0.2 V vs. RHE)	91	0.909 (22 mA cm^−2^)	[84]
3D Co SA/CC	0.5 M H_2_SO_4_	51 mA cm^−2^ (0.1 V vs. RHE)	> 80 (0.1 – 0.7 V vs. RHE)	0.676 (1.6 V vs. RHE)	[85]
Ni–SA/G–0	0.1 M KOH	2.11 A ${\mathrm{mg}}_{\mathrm{Ni}}^{-1}$ (0.60 V vs. RHE)	> 94 (0.1 – 0.5 V vs. RHE)	N/A	[86]
Fe–CNT	0.1 M KOH	10.6 (0.75 V vs. RHE)	95	N/A	[66]
Fe–CNT	0.1 M phosphate buffer solution (PBS)	6.5 (0.45 V vs. RHE)	90	∼461 mg L^−1^ h^−1^ (20 mA cm^−2^)	[66]
Pt/HSC	0.1 M HClO_4_	97.6 (0.45 V vs. RHE)	96	∼1.95	[49]
Mo_1_/OSG–H	0.1 M KOH	4.45 (0.75 V vs. RHE)	95	N/A	[87]
Mo_1_/OSG–H	PBS (pH = 10.9)	8.75 (0.60 V vs. RHE)	86	N/A	[87]
Mo_1_/OSG–H	PBS (pH = 8.7)	12.5 (0.45 V vs. RHE)	77	N/A	[87]
Ni–N_2_O_2_/C	0.1 M KOH		96	5.9 (70 mA cm^−2^)	[88]
h–Pt_1_–CuS_*x*_	0.1 M HClO_4_	8.25 (0.65 V vs. RHE), ∼72.1 (0.45 V vs. RHE)	92–96	0.546 ± 0.03	[89]
Pt/TiN	0.1 M HClO_4_	78 (0.05 V vs. RHE)	65	N/A	[90]
Pt_1_/TiN	0.1 M HClO_4_	− 0.34 mA cm^−2^ (0.2 V vs. RHE)	53.1	N/A	[91]
Pt_1_/TiC	0.1 M HClO_4_	− 0.96 mA cm^−2^ (0.2 V vs. RHE)	68	N/A	[91]
Au/TiC	0.1 M HClO_4_	4.16 A ${\mathrm{mg}}_{\mathrm{Au}}^{-1}$ (0.2 V vs. RHE)	87 (0.2 V vs. RHE)	N/A	[92]
Au_0.92_–Pd_0.08_	0.1 M HClO_4_	N/A	95	N/A	[93]
Pt–Hg	0.1 M HClO_4_	∼26 (0.65 V vs. RHE)	90	N/A	[77]
Pd–Hg	0.1 M HClO_4_	∼133 (0.65 V vs. RHE)	95	N/A	[94]
AD–Pt@AuCu–144	0.1 M HClO_4_	N/A	91.8	9.5 mg L^−1^ h^−1^ (2 mA cm^−2^)	[95]
Co–N_4_(O)–C	0.1 M KOH	∼67 (0.75 V vs. RHE)	82	0.418 ± 0.019 (50 mA)	[96]
Co–N_4_(O)–C	0.1 M PBS	∼96 (0.45 V vs. RHE)	70	N/A	[96]
0.2 wt.% Pt/TiC	0.1 M HClO_4_	N/A	75 (~ 0.075 V vs. RHE)	N/A	[97]

In view of the high cost of precious metals, SACs based on non-noble transition metals have also been investigated. Two research groups studied the activity and selectivity trends for transition metal single atoms (M SACs, M = Mn, Fe, Co, Ni, and Cu) anchored on nitrogen-doped carbon materials for electrocatalytic H_2_O_2_ production [82, 83]. Interestingly, both studies demonstrated that a Co SAC exhibited the highest catalytic activity for H_2_O_2_ synthesis via the 2e^−^–ORR pathway. This is because the Co SAC exhibited the optimum *OOH adsorption energy, according to DFT calculations (Fig. 7d). In contrast, a Ni SAC and a Cu SAC were hindered by weak *OOH adsorption. Conversely, excessively strong binding with *OOH limited the effectiveness of a Mn SAC and a Fe SAC. Sun et al. first realized an 80% selectivity for H_2_O_2_ synthesis with a Co SAC in 0.5 M H_2_SO_4_ at 0.1 V vs. RHE (Fig. 7e) [82]. Subsequently, they performed the measurement in a commercial microflow cell and reached an H_2_O_2_ yield capacity of 4.33 mol ${\mathrm{g}}_{\mathrm{cat}.}^{-1}$ h^−1^ at 50 mA cm^−2^ (Fig. 7f).

In contrast to the zeolitic imidazolate framework (ZIF)-derived SACs mentioned above, a ball milling method for forming SAC precursors was used for mechanical mixing of certain amounts of melamine, L-alanine, and transition metal acetates. Subsequently, N_2_ pyrolysis and then acid leaching were conducted to produce M–NC SACs (M = Mn, Fe, Co, Ni, and Cu). In 0.1 M HClO_4_, a Co–NC presented a high FE exceeding 90% (Fig. 7g) at 0.6 V vs. RHE. From the operando X-ray absorption spectroscopy (XAS) results shown in Fig. 7h, dynamic shifts of the active center during the electrocatalytic processes can be observed due to variations in Co–N distances [70]. When O_2_ was adsorbed on Co, the Co–N distance was elongated from 1.25 to 1.35 Å, and the Co–N distance decreased to 1.32 Å when H_2_O_2_ was produced at Co sites. Simultaneously, the rate-determining step for H_2_O_2_ production at Co–NC shifted from * + O_2_ + e^−^  → *O_2_ (step 1) to * + O_2_ + H^+^  + e^−^  → *OOH (step 2) with increasing reaction potential (Fig. 7i).

**Fig. 8:**
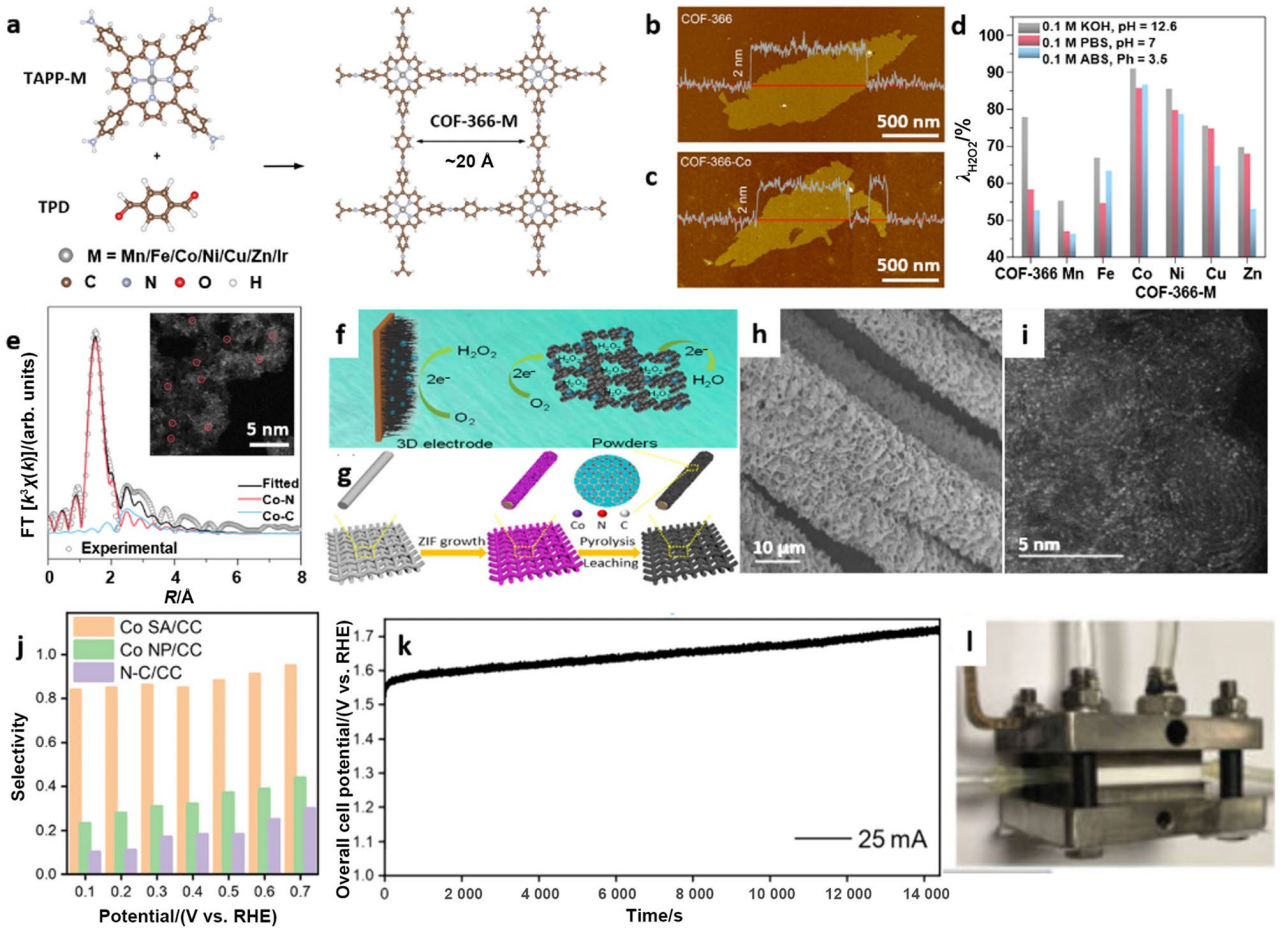
**a** Schematic illustration of the synthesis of COF–366–M, 1 Å = 1×10^−10^ m. Atomic force microscopy (AFM) images of **b** COF–366 and **c** COF–366–Co and the corresponding height profiles along the red lines.** d** H_2_O_2_ selectivity for three electrolytes with COF–366–M. **e** Fitted Co K-edge EXAFS spectrum of COF–366–Co. Panels (**a–e**) are reprinted with permission from Ref. [84]. Copyright © 2020, American Chemical Society. **f** Schematic illustration showing a comparison between a three-dimensional (3D) electrode and an electrode built from catalyst powders for the ORR. **g** Schematic illustration showing the preparation of a hierarchical, free-standing electrode. **h** Scanning electron microscopy (SEM) image of Co SA/CC. **i** HAADF-STEM image of Co SA/CC. **j** H_2_O_2_ selectivity of Co nanoparticles (NP)/CC, Co SA/CC, and N–C/CC obtained in 0.5 M H_2_SO_4_. **k** Operating curve for H_2_O_2_ production in a commercial flow cell. **l** Digital photograph of the H_2_O_2_-producing flow cell. Panels (**f–l**) are reprinted with permission from Ref. [85]. Copyright © 2020, John Wiley and Sons

In addition to carbon-supported SACs, covalent organic framework (COF)-based materials with various intrinsically isolated metal centers have also proven to be promising candidates for electrocatalytic H_2_O_2_ production via the 2e^−^ ORR. For instance, a series of COF–366–M SACs (M = Mn, Fe, Co, Ni, Cu, and Zn) were synthesized by the imine condensation process occurring between various 3d-transition metal ions loaded as 5,10,15,20-(tetra-4-aminophenyl)porphyrin salts (TAPP-M, M = Mn, Fe, Co, Ni, Cu, and Zn) and terephthaldehyde (TPD) during a solvothermal reaction (Fig. 8a), which resulted in uniform two-dimensional (2D) structures (Fig. 8b, c) [84]. Thus, different metal active sites were successfully incorporated into porphyrin moieties with identical active site densities. Among them, COF–366–Co exhibited the highest selectivity of 91% in 0.1 M KOH (Fig. 8d) and a yield of 909 mmol h^−1^, which can be attributed to the specially designed Co–N–C active sites (Fig. 8e). DFT calculations further confirmed that the high activity and selectivity of this material for H_2_O_2_ generation resulted from the initial adsorption of O_2_ as well as the stability of HOOH* species on active sites. Overall, catalytically active Co–N–C sites convert O_2_ to O_2_* and bind both O_2_* and HOOH* species with appropriate strengths that are neither too strong nor too weak, leading to optimal performance in electrocatalytic H_2_O_2_ formation via 2e^−^–ORR.

Furthermore, a three-dimensional (3D) and free-standing Co-based SAC electrode (Fig. 8f) was developed as a binder-free electrocatalyst for H_2_O_2_ production [85]. For this purpose, a nanoflake Co single-atom electrode (Co SA/CC, Fig. 8g–i) was fabricated by pyrolyzing a Co-containing ZIF grown on carbon cloth (CC). In 0.5 M H_2_SO_4_, a selectivity of over 80% was achieved within a potential range of 0.1 to 0.7 V vs. RHE (Fig. 8j). Using this catalyst, an H_2_O_2_ concentration of 1 840 mg L^−1^ (0.676 mol ${\mathrm{g}}_{\mathrm{cat}.}^{-1}$ h^−1^) was obtained after 4 h of electrocatalysis in a flow cell (Fig. 8k, l) at a potential of 1.6 V vs. RHE. Apart from the excellent catalytic performance, the 3D free-standing Co SAC electrode also precluded the use of binders, which not only improved the electrical conductivity to some extent but also eliminated the detachment of catalyst seen with conventional electrodes.

**Fig. 9:**
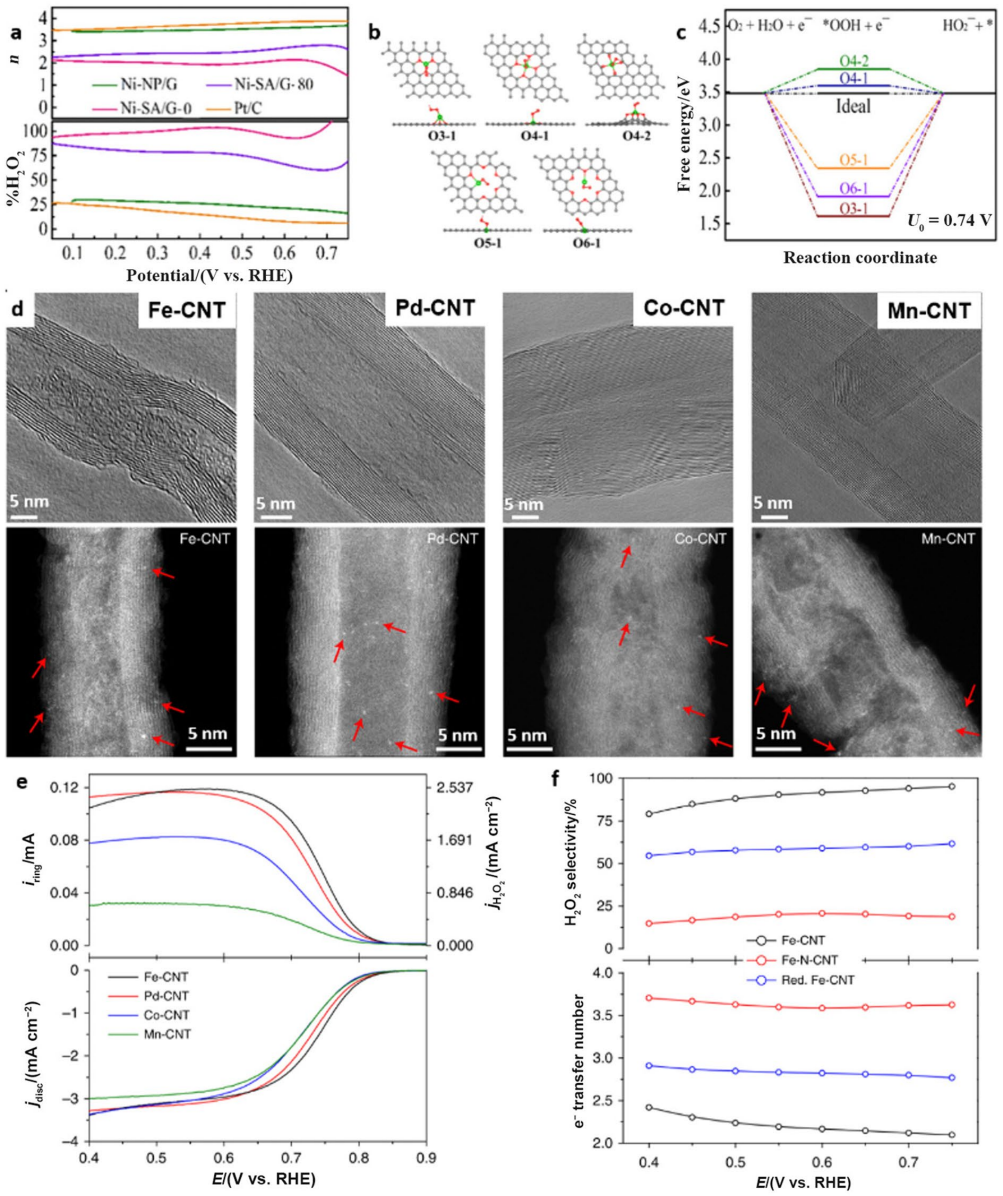
**a** Electron transfer number and H_2_O_2_ selectivity of Ni–SA/G–0, Ni–SA/G–80, Ni–NP/G, and commercial Pt/C catalysts. **b** Top and side views of the configurations for *OOH adsorption (O3-1, O4-1, O4-2, O5-1, and O6-1). Red, gray, pink, and green balls represent O, C, H, and Ni atoms, respectively. **c** Calculated free-energy diagram for the production of HO_2_^−^ at pH = 13.0. Panels (**a–c**) are reprinted with permission from Ref. [86]. Copyright © 2020, American Chemical Society. **d** High-resolution transmission electron microscopy (HRTEM) and aberration-corrected HAADF-STEM images of M–CNTs (M = Fe, Pd, Co, Mn). Bright dots in HAADF-STEM images indicated by red arrows represent single metal atoms. **e** Linear sweep voltammetry of the CNT background and M–CNT (M = Fe, Pd, Co, Mn) catalysts in 0.1 M KOH. **f** Calculated H_2_O_2_ selectivity and electron transfer number during a potential sweep with M–CNT (M = Fe, Pd, Co, Mn) catalysts. Reprinted with permission from Ref. [66]. Copyright © 2019, Springer Nature

Along with the rapid advancement of Co-based SACs toward practical application for H_2_O_2_ synthesis, other materials, such as Ni-based SACs, have also shown feasibility under alkaline conditions. For example, a Ni single-atom catalyst (i.e., Ni–SA/G–0) was synthesized on graphene by reducing a mixture of dimethyl formamide, NiCl_2_, and graphite oxide with NaBH_4_; the resulting material catalyzed 2e^−^ H_2_O_2_ production in 0.1 M KOH with a selectivity higher than 94% at potentials ranging from 0.1 to 0.5 V vs. RHE (Fig. 9a) [86]. Five possible binding models were proposed and assessed to identify the mechanism by which the highly active Ni–SA/G–0 reduced oxygen to generate H_2_O_2_ (Fig. 9b). As shown in Fig. 9c, Ni–O_4_–C was the optimal coordination figuration, while the O4-1 model required the smallest overpotential to form *OOH.

#### Regulation of Coordination Environment

After the selection of active metal centers, another challenge in fabricating SACs for H_2_O_2_ formation via the 2e^−^–ORR is to further tune the catalytic mechanisms of these single-atom active sites by using appropriate coordination regulation strategies. To date, a variety of strategies have been proposed to regulate the coordination environment of single-atom centers in terms of coordination binding strength and atomic dispersive durability [49, 98–100]. Specifically, by modifying the neighboring coordination atoms, support coupling conditions, and functional groups, both the electronic and geometric structures of isolated metal sites can be altered, and the selectivity of SACs for H_2_O_2_ formation via the 2e^−^ ORR path would be fully adjustable. The resulting anchoring sites could also stabilize the single metal atom centers during fabrication of the materials and throughout their catalytic processes [40, 101].

##### Modification of Neighboring Coordinating Atoms

Numerous studies on the coordination structures of single-atom sites have suggested that isolated M–X–C (X = N, O, S,…) species are active in various electrocatalytic reactions [102, 103]. After the selection of metal atom centers, different ligand atoms on the surface can modify the intrinsic adsorption strengths of various intermediates to generate targeted electrocatalytic activity. On the basis of previous investigations, Fe–N_4_ is generally recognized as a highly efficient active site for electrocatalytic ORR via the 4e^−^ route, and its activity is even comparable to those of state-of-the-art commercial Pt/C catalysts [104, 105]. Nevertheless, when Fe atoms are coordinated with carbon and oxygen to form the Fe–C–O configuration, the ORR product turns from H_2_O to H_2_O_2_, as confirmed with a series of CNT-supported single-atom catalysts containing transition metal centers (i.e., M–CNT, M = Fe, Pd, Co, Mn, Fig. 9d) [66]. Experimentally, Fe–CNTs demonstrated high activity (onset potential: 0.822 V vs. RHE, Fig. 9e) and selectivity (95%) in 0.1 M KOH (Fig. 9f), which can be ascribed to the well-designed Fe–C–O active sites. Consequently, high activity and selectivity can be modulated by controlling the single-atomic metal centers and nearby coordination environments.

**Fig. 10:**
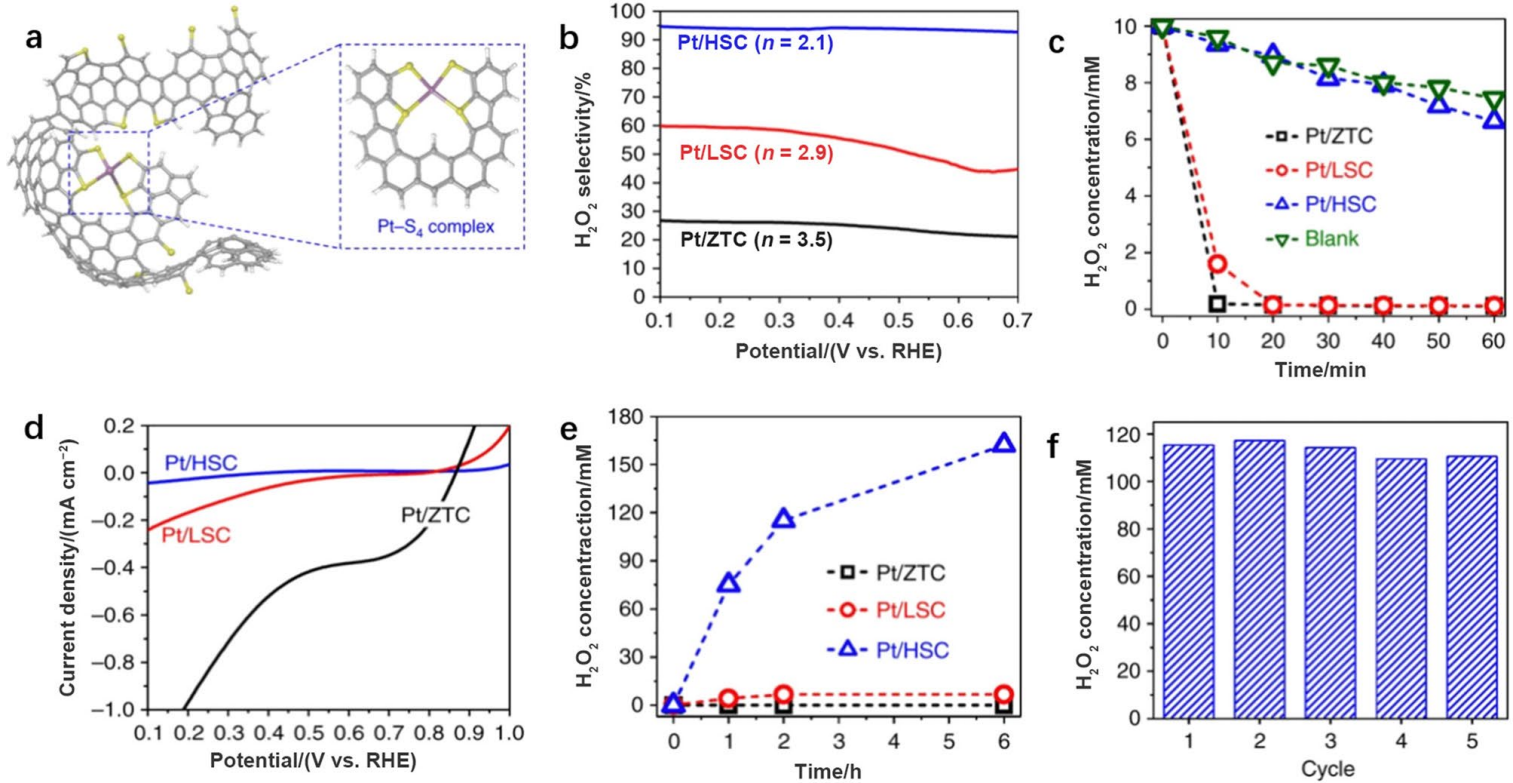
**a** Proposed structure of Pt/HSC with a Pt–S_4_ coordination environment (C, gray; H, white; S, yellow; and Pt, purple). **b** H_2_O_2_ production selectivity of Pt/HSC, Pt/LSC, and Pt/ZTC in 0.1 M HClO_4_. **c** Peroxide disproportionation reaction activities resulting in the decline in H_2_O_2_ concentration in a 10 mM H_2_O_2_ starting solution (50 mL) with 10 mg of catalysts. **d** Peroxide reduction activities tested in 10 mM H_2_O_2_/0.1 M HClO_4_. **e** Accumulated H_2_O_2_ concentrations in an H-cell with 1.0 M HClO_4_ electrolyte and a Nafion 115 membrane. The H-cell was operated under short-circuit conditions (*V* = 0) at 278 K. **f** Concentrations of H_2_O_2_ produced on Pt/HSC in an electrochemical H-cell during repeated 2 h operation cycles. Reprinted with permission from Ref. [49]. Copyright © 2016, Springer Nature

Pt, which is known to catalyze the 4e^−^ ORR, can also be tuned to produce H_2_O_2_ selectively when single Pt atoms are coordinated with S to form Pt–S_4_–C (Fig. 10a). For example, Pt single atoms (5 wt.%) were anchored on carbon supports with a high S contents (Pt/HSC) via a zeolite template method, and the system exhibited high selectivity for H_2_O_2_ production (96%) in 0.1 M HClO_4_ (Fig. 10b) [49]. Since the H_2_O_2_ product is easily reduced, a good electrocatalyst for H_2_O_2_ should also limit the decomposition of H_2_O_2_. Compared with Pt clusters or nanoparticles supported on carbon, Pt/HSC showed reduced activity for H_2_O_2_ decomposition (Fig. 10c, d), which was mainly attributed to the highly isolated Pt single atoms and modulation by the nearby S ligands. A fuel cell was built using this Pt/HSC catalyst, and it generated a H_2_O_2_ concentration of 160 mM after 6 h of reaction (Fig. 10e) and also generated electricity. After a 2-h cycle, Pt/HSC still exhibited good stability (Fig. 10f) with little attenuation of H_2_O_2_ concentration, further confirming the importance of neighboring atoms for adjusting selectivity.

**Fig. 11:**
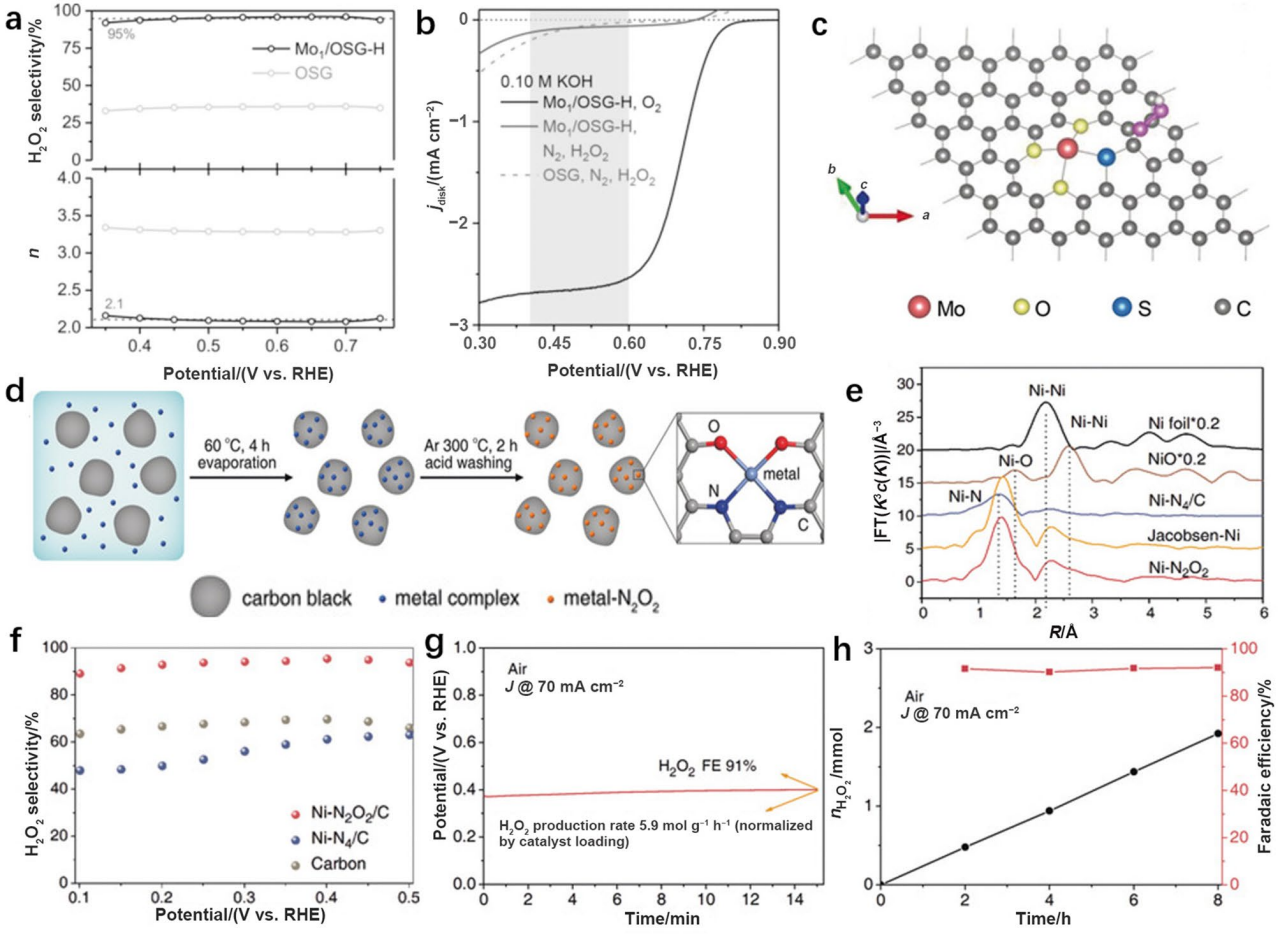
**a** Calculated electron transfer number and H_2_O_2_ selectivity of Mo_1_/OSG–H and OSG catalysts via 2e^−^ ORR in 0.1 M KOH. **b** Linear sweep voltammetry (LSV) curves for H_2_O_2_ reduction in N_2_-saturated 0.1 M KOH containing 10 mM H_2_O_2_. **c** Atomic configuration of OOH* adsorption on Mo–O_3_S–C. Panels (**a–c**) are reprinted with permission from Ref. [87]. Copyright © 2020, John Wiley and Sons. **d** Schematic illustration of the synthesis of Ni–N_2_O_2_/C. **e** k^2^-weighted Fourier transforms of the Ni K-edge EXAFS spectra for Ni–N_2_O_2_/C, Jacobsen–Ni, Ni–N_4_/C, and various nickel reference materials. **f** H_2_O_2_ selectivity of Ni–N_2_O_2_/C, Ni–N_4_/C, and carbon catalysts in 0.1 M KOH. **g** H_2_O_2_ concentration and the chronopotentiometry curve measured in a flow cell in the presence of air. **h** Concentration of H_2_O_2_ produced (left) and H_2_O_2_ FE (right) of a Ni–N_2_O_2_/C catalyst tested for 8 h in the flow cell in the presence of air. Panels (**d–h**) are reprinted with permission from Ref. [88]. Copyright © 2020, John Wiley and Sons

In addition to metal atom coordination spheres containing only one type of element, coordination spheres comprising different elements provide another way to regulate the catalytic properties of SACs. It is important to tune the type, quantity, and variety of coordination sites to achieve maximal loading of single metal atoms and stimulate higher activities. For example, Mo single atoms were coordinated with O and S donors to form a SAC with 10 wt.% loading of Mo (denoted as Mo_1_/OSG–H); this system showed a high selectivity (95%) for H_2_O_2_ generation in 0.1 M KOH over a wide potential range of 0.45 to 0.60 V vs. RHE (Fig. 11a) [87]. Compared with O/S-dual coordinated graphene (OSG), OSG containing 6.89 wt.% Mo (Mo_1_/OSG–M) and 0.21 wt.% Mo (Mo_1_/OSG–L), Mo_1_/OSG–H with appropriate Mo–O/S–C active sites exhibited the best capacity for preventing further reduction in H_2_O_2_ (Fig. 11b). DFT calculations further revealed that the unique coordination structure (i.e., Mo–O_3_S–C, Fig. 11c) greatly impacted *OOH adsorption, leading to catalysis via the desired 2e^−^–ORR pathway. Similarly, O,N-dual coordinated Ni centers were anchored on carbon black Ni–N_2_O_2_/C, which was fabricated by pyrolyzing Ni-coordinated Jacobsen’s ligand (Fig. 11d) [88]. On this material, a Ni–N_2_O_2_ coordination sphere similar to the Ni species in the Jacobsen–Ni complex was successfully produced (Fig. 11e). In 0.1 M KOH, a H_2_O_2_ selectivity of 96% was realized with Ni–N_2_O_2_/C with an average electron transfer number of *n* = 2.09 at 0.4 V vs. RHE (Fig. 11f). Furthermore, an H_2_O_2_ yield rate of 5.9 mol  ${\mathrm{g}}_{\mathrm{cat}.}^{-1}$  h^−1^ (Fig. 11g) was reached at 70 mA cm^−2^ in a three-phase flow cell exhibiting stable operation over a period of 8 h (Fig. 11h). Thus, compared to conventional Ni–N_4_ coordination, the electrocatalytic H_2_O_2_-producing properties of Ni SACs via the 2e^−^ ORR were improved by building the Ni–N_2_O_2_ coordination configuration.

##### Support Coupling Modification

**Fig. 12:**
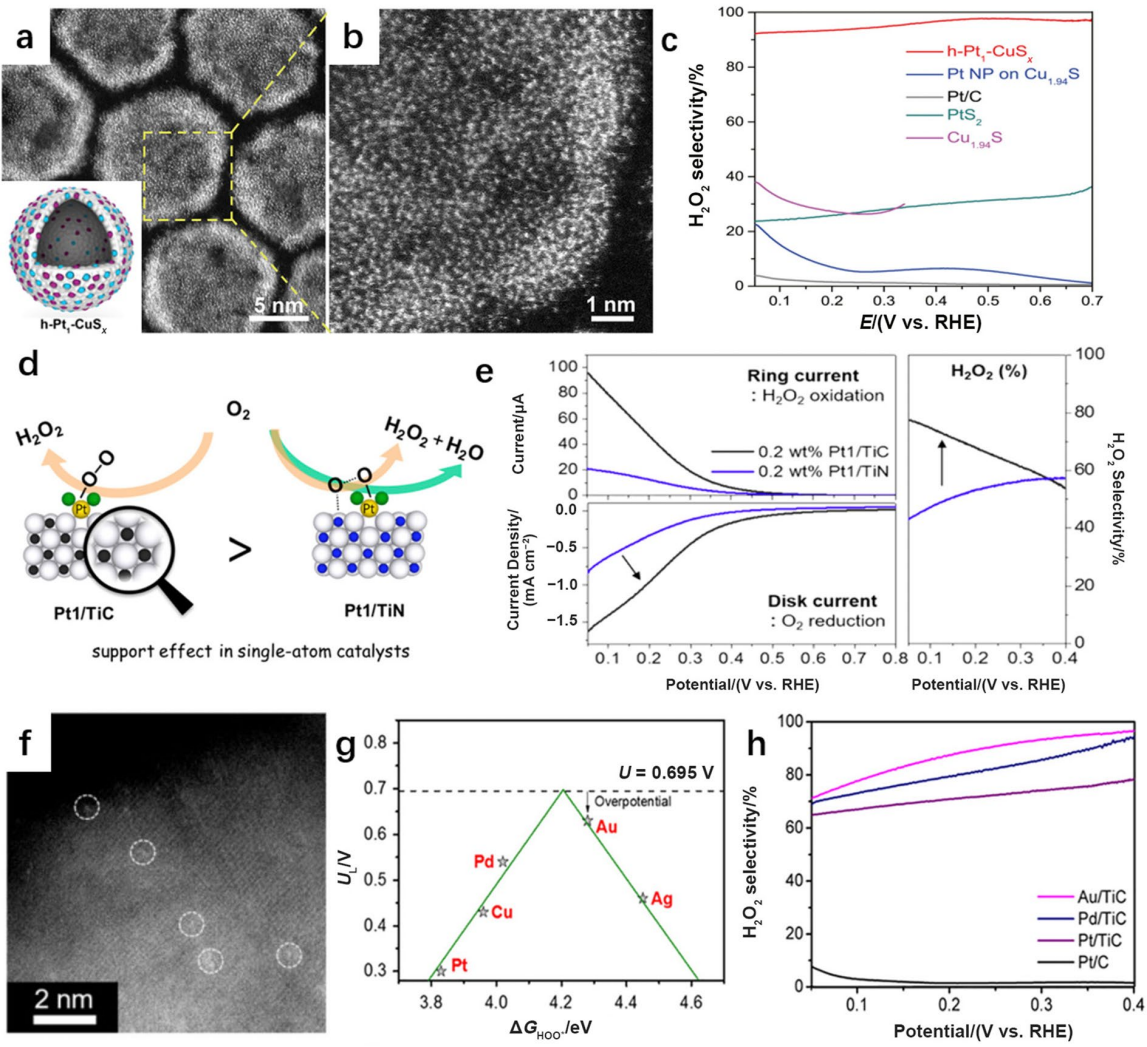
**a** HAADF-STEM images of h–Pt_1_–CuS_*x*_. Insert image shows the structure of h–Pt_1_–CuS_*x*_. Blue, purple, and white balls represent Cu, Pt, and S atoms, respectively. **b** HAADF-STEM images of h–Pt_1_–CuS_*x*_. **c** H_2_O_2_ selectivity of the h–Pt_1_–CuS_*x*_ catalyst in 0.1 M HClO_4_. Panels (**a–c**) are reprinted with permission from Ref. [89]. Copyright © 2019, Elsevier. **d** Schematic illustration of support effect in SACs. **e** ORR polarization curves (left) and H_2_O_2_ selectivity (right) measured in O_2_-saturated 0.1 M HClO_4_ solution. Panels (**d, e**) are reprinted with permission from Ref. [91]. Copyright © 2017, American Chemical Society. **f** HAADF-STEM image of Au/TiC (circles: Au atoms). **g** Volcano plot of the ORR for the limiting potential of M/TiC (M = Cu, Ag, Au, Pd, and Pt) with Δ*G*_HOO*_. **h** H_2_O_2_ selectivity of SACs and commercial Pt/C measured in O_2_-purged 0.1 M HClO_4_ solution. Panels (**f–h**) are reprinted with permission from Ref. [92]. Copyright © 2019, American Chemical Society

The coordination modification strategy introduced above is mainly adopted with carbon-based supports, which possess versatile surface features for modifying the electrocatalytic properties of SACs. In addition, metal compounds (e.g., oxides [106], chalcogenides [89], carbides [92], and nitrides [90]), with metal atoms exposed on their surfaces and a variety of unsaturated sites, are recognized as support materials suitable for stabilizing isolated metal sites with strong chemical bonds [79]. For instance, by utilizing the strong interaction between Pt and S, a high loading of atomically dispersed Pt (~ 24.8 at%, at% means in the atomic percentage) on hollow CuS_*x*_ nanospheres (h–Pt_1_–CuS_*x*_, Fig. 12a, b) was obtained [89]. Homogeneously dispersed Pt active sites and high loadings led to the enhancement of selectivity and activity for H_2_O_2_ production. Consequently, H_2_O_2_ was continuously generated in 0.1 M HClO_4_ with high selectivity of 92%–96% at potentials ranging from 0.05 to 0.70 V vs. RHE (Fig. 12c).

TiN and TiC, with high electrical conductivity and strong corrosion resistance, have also been utilized as substrates for single atoms and used for electrocatalytic H_2_O_2_ production. For example, TiN-supported single-atom Pt (0.35 wt.% Pt/TiN) was fabricated by a wet impregnation method. Unlike TiN-supported Pt nanoparticles, which produced H_2_O via the 4e^−^–ORR pathway, 0.35 wt.% Pt/TiN with homogeneous distribution of Pt single atoms on the TiN surface showed a high selectivity of 65% for H_2_O_2_ synthesis at 0.04 V vs. RHE in 0.1 M HClO_4_ [90]. Additionally, TiC-supported single-atom Pt (Pt_1_/TiC) was also fabricated with a similar wet impregnation method for H_2_O_2_ synthesis (Fig. 12d) [91]. In comparison with the TiN-supported counterpart (i.e., Pt_1_/TiN), the selectivity of Pt_1_/TiC in 0.1 M HClO_4_ was 68% at 0.1 V vs. RHE, which was greater than that of Pt_1_/TiN (53.1%) (Fig. 12e). DFT calculations revealed that the weaker adsorption of *OOH on Pt_1_/TiC facilitated the release of active sites, leading to better catalytic activity and selectivity. Consequently, instead of the well-known supporting function of stabilizing single atoms, these supports participated in the reactions and had multiple functions in the construction of SACs.

To systematically evaluate electrocatalytic H_2_O_2_ production via the 2e^−^ ORR on TiC-supported SACs, various transition single-atom metal candidates (M/TiC, M = Cu, Ag, Au, Pd, and Pt) were screened [92]. Theoretically, Au/TiC (Fig. 12f) stands out because it showed lowest overpotential in the volcano plot (Fig. 12g). Experimentally, the highest selectivity (87% at 0.2 V vs. RHE) of Au/TiC in 0.1 M HClO_4_ also exceeded those of other metals. Thus, the selections of isolated metal centers and supports are essential for modulating the electronic and geometric effects of SACs, and they are equally significant for modifying the behavior of SACs in electrocatalytic H_2_O_2_ production.

**Fig. 13:**
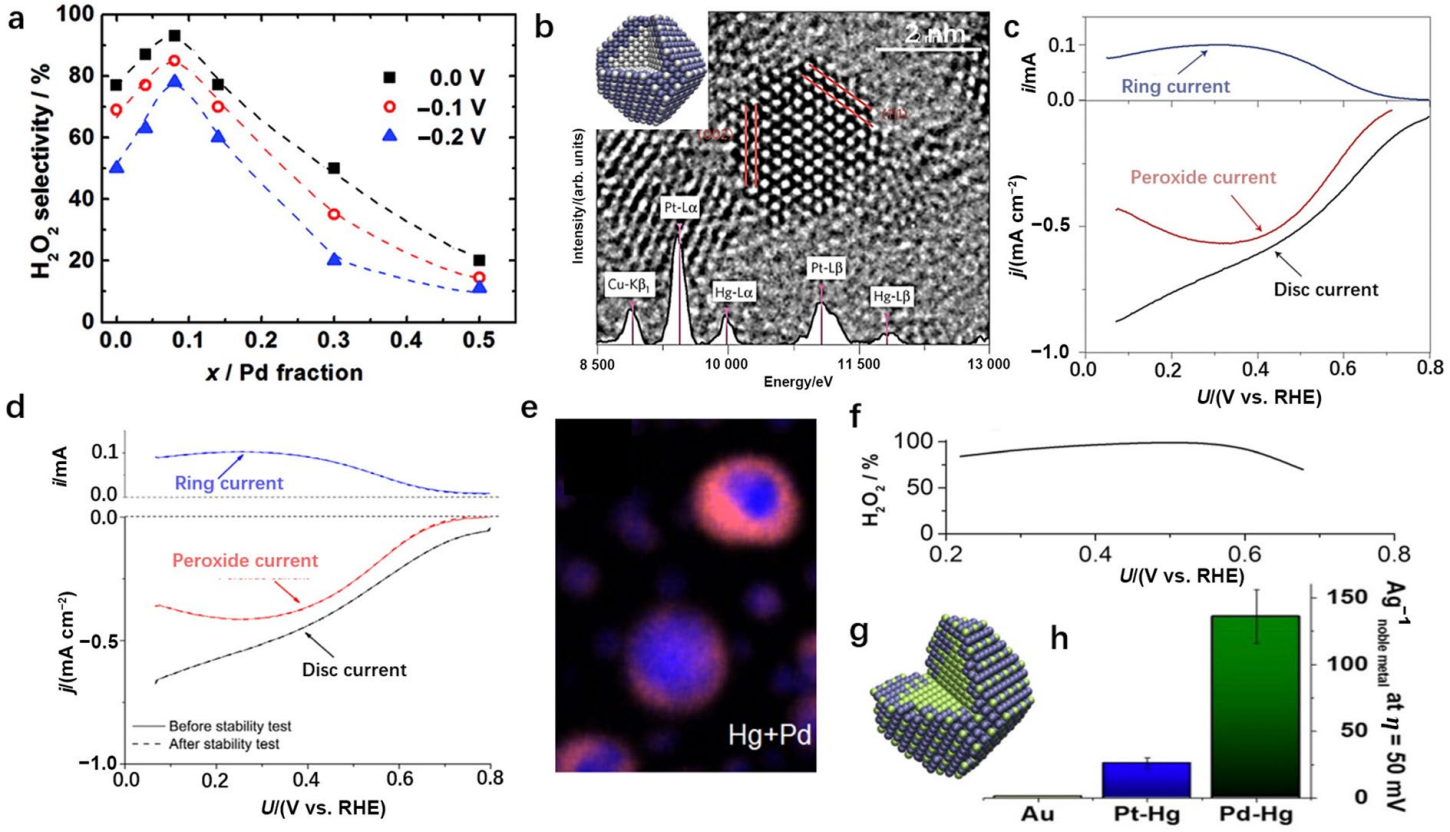
**a** ORR selectivity for H_2_O_2_ production as a function of palladium content, *x*, at potentials of 0 V (squares), − 0.1 V (circles), and − 0.2 V (triangles). The dashed lines were added to guide the eye. Reprinted with permission from Ref. [93]. Copyright © 2011, American Chemical Society. **b** HRTEM image of a single Pt–Hg alloy, with the corresponding energy dispersive spectroscopy (EDS) spectrum of the particle superimposed on top. The insert contains a schematic representation of the Pt–Hg alloy; blue, mercury; grey, platinum. **c** LSV of Pt–Hg alloy tested in O_2_-saturated 0.1 M HClO_4_ electrolyte with the disc current, ring current, and current corresponding to hydrogen peroxide obtained from the ring current. **d** Stability test for Pt–Hg/C consisting of 8 000 cycles between 0.05 and 0.8 V in O_2_-saturated 0.1 M HClO_4_. Panels (**b–d**) are reprinted with permission from Ref. [77]. Copyright © 2013, Springer Nature. **e** STEM-EDS elemental mapping of Hg + Pd in the Pd–Hg catalyst. **f** H_2_O_2_ selectivity of the Pd–Hg catalyst measured in 0.1 M HClO_4_. **g** Schematic representation of a Pd–Hg alloy with Pd colored green and Hg colored blue. **h** Mass activity (A g^−1^ of noble metal) at 50 mV overpotential for Pd–Hg, Pt–Hg alloy, and Au. Panels (**e–h**) are reprinted with permission from Ref. [94]. Copyright © 2014, American Chemical Society

Because of metal–metal interactions among the isolated metal atoms and alloy substrates, improved electrocatalytic performance was achieved with SAAs [107]. Jirkovský et al. developed a Au_0.92_–Pd_0.08_ SAA electrocatalyst with single Pd atoms anchored on the gold surface [93]. By treating a mixed aqueous solution of HAuCl_4_ and poly(vinyl alcohol) (PVA) with NaBH_4_ reductant, a Au colloidal solution was obtained; this was mixed with carbon black for subsequently annealing under an atmosphere containing 10 vol% H_2_ and 90 vol% Ar (vol% means the volume percentage). Then, PdCl_2_ solution was added to these Au-carbon black solid mixtures. In this way, Au_0.92_–Pd_0.08_ SAA was fabricated by bubbling H_2_ for 1 h. It turned out that Au_0.92_–Pd_0.08_ SAA produced a selectivity of 95% for H_2_O_2_ generation via the 2e^−^ ORR at 0 V vs. RHE in 0.1 M HClO_4_ (Fig. 13a), which could be attributed to strong interactions between the individual surface Pd atoms and the surrounding Au atoms. After that, Siahrostami et al. reported a Pt–Hg SAA, which was fabricated by electrodepositing Hg from a HgClO_4_ solution on a polycrystalline Pt disc. The as-obtained Pt–Hg SAA presented a Pt core and isolated Pt atoms dispersed on the surface of Hg shell (Fig. 13b) [77]. A selectivity higher than 90% was achieved in 0.1 M HClO_4_ at potentials ranging from 0.3 to 0.5 V versus RHE (Fig. 13c). Significantly, no noticeable attenuation of H_2_O_2_ formation was observed after 8 000 potential cycles (Fig. 13d), suggesting the extremely high stability of this SAA catalyst. Hence, the presence of atomically discrete Pt active centers on the inert Hg shell optimized the adsorption of oxygen to avoid O–O bond breaking. A similar concept was also verified for Pt–Au and Pd–Hg SAA catalysts [94, 108]. For example, Stephens and coworkers developed a Pd–Hg SAA with a Pd core and a matrix comprising isolated Pd atoms dispersed in a Hg shell (Fig. 13e, g). From 0.35 to 0.55 V versus RHE, the Pd–Hg SAA exhibited a selectivity for H_2_O_2_ generation of over 95% in 0.1 M HClO_4_, further verifying the concept proposed above (Fig. 13f) [94]. More importantly, the mass activity of the Pd–Hg SAA exceeded even that of the Pt–Hg analog by a factor of five (Fig. 13g).

**Fig. 14:**
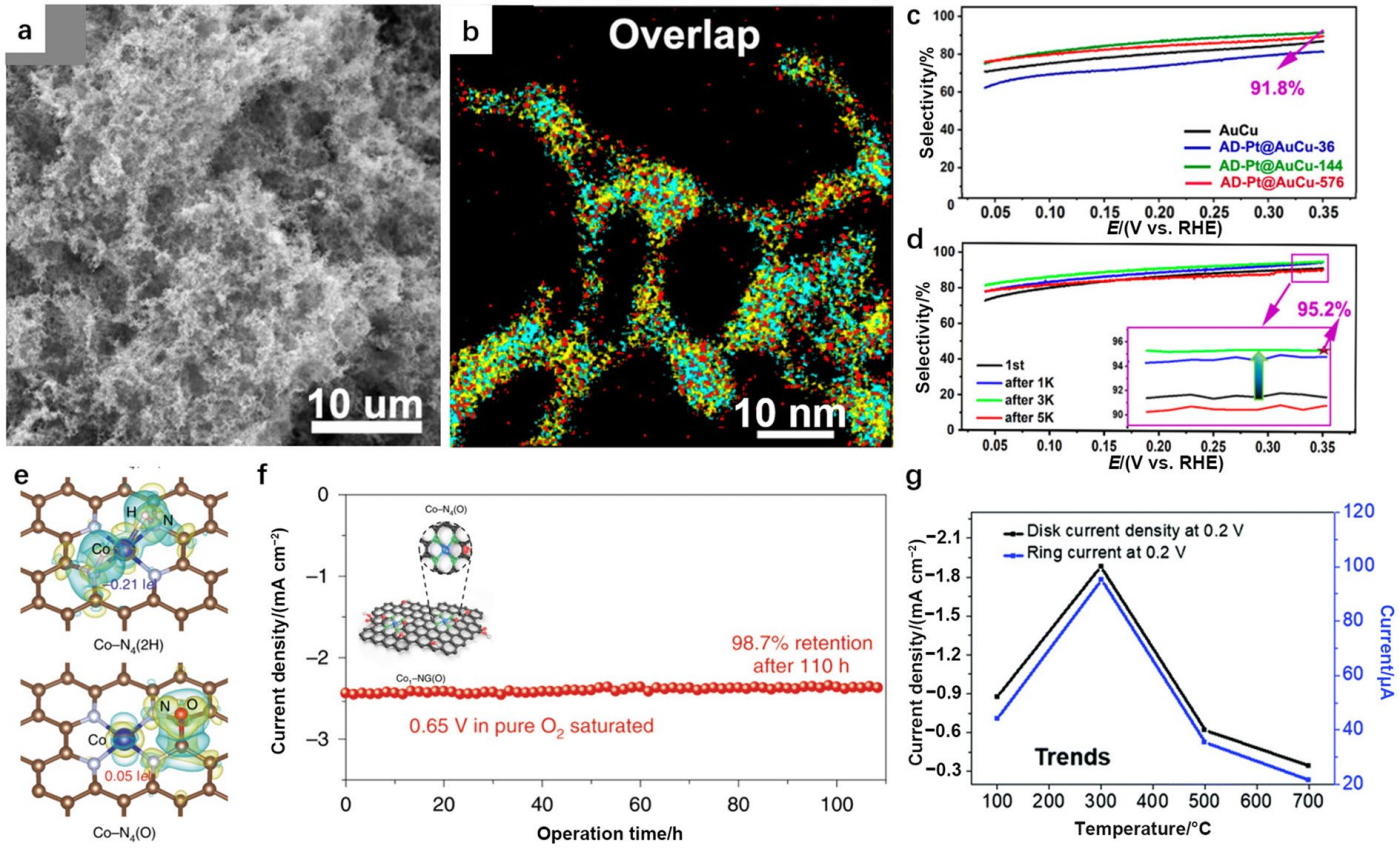
**a** SEM image of AD–Pt@AuCu–144 hydrogels. **b** HAADF-STEM-EDS mapping overlap image of AD–Pt@AuCu–144 hydrogels. **c** H_2_O_2_ selectivity of AuCu and AD–Pt@AuCu–*x* (*x* = 36, 144, and 576) catalysts measured in O_2_-saturated 0.1 M HClO_4_ electrolyte. **d** H_2_O_2_ selectivity (upper inset; zoomed-out image at 0.35 V vs. RHE) of the AD–Pt@AuCu–144 catalyst after the accelerated durability test (ADT). Panels (**a–d**) are reprinted with permission from Ref. [95]. Copyright © 2019, American Chemical Society. **e** Differential charge densities of Co–N_4_/graphene after adsorption of 2H* or O* near the cobalt atom. Yellow and cyan isosurfaces show electron gain and electron loss, respectively. **f** Chronoamperometry stability test of Co_1_–NG(O) at 0.65 V in 0.1 M KOH. The inset shows a schematic diagram of the Co_1_–NG(O) SAC and the Co–N_4_(O) structure. Panels (**e–g**) are reprinted with permission from Ref. [96]. Copyright © 2020, Springer Nature. **g** Disk currents for 0.2 wt.% Pt/TiC catalysts with various reduction temperatures measured in O_2_-saturated 0.1 M HClO_4_ solution. Reprinted with permission from Ref. [97]. Copyright © 2019, Royal Society of Chemistry

Compared to those of monometallic substrates, the synergistic effects induced by multimetallic alloy supports would be more conducive to adjustments of the electrocatalytic performance of SAAs. Shi et al. selected AuCu nanoaerogels to load single Pt atoms with a galvanic replacement reaction using H_2_PtCl_6_ solution as a Pt precursor, and this resulted in atomic Pt dispersed on the AuCu alloy (AD–Pt@AuCu–144, Fig. 14a, b) [95]. As shown in Fig. 14c, an H_2_O_2_ selectivity of 91.8% and an obvious selectivity enhancement were observed in 0.1 M HClO_4_ during 3 000 to 5 000 cycles (Fig. 14d). This phenomenon was attributed to rearrangements involving the dispersed Pt atoms and the robust 3D porous structure of the AuCu alloy. Thus, the employment of multimetallic alloy supports allows further tuning of the electronic and geometric structures of active sites for high-efficiency H_2_O_2_ production.

##### Functional Group Modification

Incorporating functional groups with active moieties in SACs would offer more opportunities for precisely modifying the binding between active sites and intermediate products during H_2_O_2_ formation, resulting in the careful regulation of catalytic performance. Li et al. discovered that atomic Co–N_*x*_–C active sites cooperating with oxygen functional groups simultaneously boosted the activity and selectivity for producing H_2_O_2_ in alkaline electrolyte solution [109]. Furthermore, Jung et al. discovered that attaching functional groups near Co–N_4_ sites adjusted the energy for adsorption of intermediate *OOH at Co–N_4_ sites, resulting in modifications of catalytic activity and selectivity of the material [96]. The involvement of electron-rich functional groups (e.g., O*, *OH) increased ∆G_*OOH_, while electron-poor functional groups (e.g., H*, C) will caused decreases in ∆G_*OOH_. Specifically, attaching O* near Co atoms positively alter their charges by 0.05 |e| (Fig. 14e), while attaching 2H* negatively tuned the charges by 0.21 |e|. As a result, a relatively stable mass activity of (155 ± 6) A g^−1^ was achieved at 0.65 V vs. RHE for over 110 h with Co–N_4_(O) in 0.1 M KOH (Fig. 14f).

Moreover, anchoring halogen-based functional groups near the isolated metal centers of SACs also modulated their catalytic properties. For example, Shin et al. demonstrated that optimized placement of chloride ligands triggered appropriate adsorption of *OOH intermediates at Pt active sites, thus leading to optimized catalytic performance [97]. Consequently, 0.2 wt.% loading of Pt/TiC and a modest Cl/Pt atomic ratio was obtained by reduction at 300 °C (Fig. 14g), and the resulting material exhibited the best activity for H_2_O_2_ production. Therefore, the attachment of different functional groups modulates the electronic structures of isolated metal centers and makes a large impact on the electrocatalytic behavior of SACs.

## Measurements of Impacts on H_2_O_2_-Producing Electrocatalysts

### Electrolytes

Electrolytes play a critical role in electrochemical processes. Specifically, the pH, concentration of electrolytes, and types of solvents can collaboratively affect the efficiency and yield rate of electrochemical H_2_O_2_ generation via the 2e^−^ ORR route as well as the purity of the H_2_O_2_ produced. Although a high electrolyte concentration guarantees low charge transfer resistance, the concern over pollution caused by the species in the electrolyte solution also increases [110]. Thus, the use of an appropriate electrolyte concentration is very important. It is highly likely that a low pH is preferable for H_2_O_2_ production, as the hydrogenation of O_2_ accompanies the formation of H_2_O_2_. As a result, HClO_4_ and H_2_SO_4_ have been commonly used as acidic electrolytes [82, 83]. However, the competitive hydrogen evolution reaction at the cathode may also be facilitated by the increased proton concentration, resulting in a decrease in the H_2_O_2_ yield rate and FE. In alkaline conditions (e.g., with KOH as electrolytes and pH ˃ 9), H_2_O_2_ decomposes quickly upon accumulation of HO_2_^−^ (H_2_O_2_ + HO_2_^−^  → H_2_O + O_2_ + OH^−^) [20, 66, 109]. In addition, neutral electrolytes containing alkali metal salts (e.g., Na_2_SO_4_ and K_2_SO_4_) have also been utilized in H_2_O_2_ production [82, 111]. Moreover, those metal salts can also act as supporting electrolytes and boost the selectivity and activity of H_2_O_2_ production in the 2e^−^ ORR. It was found that an increase from 0.01 M to 0.05 M in the concentration of Na_2_SO_4_ supporting electrolyte in an acidic electrolyte solution with pH = 3 led to significantly increased H_2_O_2_ production, which might be attributable to enhanced electrical conductivity of the electrolyte [112].

In addition, it was also found that air-annealed glassy carbon was only capable of delivering high activity for 2e^−^ H_2_O_2_ production in 0.1 M KOH electrolyte [113], rather than with other, acidic electrolytes [83, 114, 115]. For SACs with carbonaceous material serving as the substrate, the influence of electrolyte pH also showed the same trend. For example, a series of SACs with transition metal (Mn, Fe, Co, Ni, and Cu) atoms anchored on N-doped graphene were exploited for H_2_O_2_ production via the 2e^−^ ORR [83]. It was found that these catalysts exhibited higher catalytic activity in 0.1 M KOH (pH = 13) than in 0.1 M HClO_4_ (pH = 1.2). The pH dependence of an SAC (i.e., Co–N–C) within different voltage ranges was also investigated [82]. In an alkaline (0.1 M KOH) medium, this SAC presented an onset potential of 0.95 V vs. RHE and showed an activity higher than those seen with neutral (0.1 M K_2_SO_4_, pH = 7, 0.83 V vs. RHE) or acidic (0.5 M H_2_SO_4_, pH = 0.3, 0.71 V vs. RHE) electrolyte solutions. However, in 0.5 M H_2_SO_4_, the selectivity of the catalyst for H_2_O_2_ production via the 2e^−^ ORR increased with a negative shift in potential from 0.5 V to 0.1 V vs. RHE. In neutral and alkaline media, negligible changes were found for selectivity at different testing potentials. This might be due to the influence of pH on surface functional groups and coordination between single atoms and substrates, which could lead to variations in the energy for binding of catalysts and oxygen molecules. In general, weak binding of oxygen at the catalyst surface leads to strong dependence of electrocatalytic performance on the pH of the electrolyte [7].

### Effects of Oxygen Input and Reaction Temperature

Typically, use of the electrocatalytic 2e^−^ ORR for production of H_2_O_2_ is conducted in an O_2_-saturated electrolyte at room temperature. In this case, the purity and flow rate of O_2_ are important factors impacting the efficiency of H_2_O_2_ production. High oxygen purity and flow rate directly enhance H_2_O_2_ electrosynthesis [20]. To further improve the fraction of oxygen utilized for H_2_O_2_ production (%, Eq. 4), gas diffusion electrodes (GDEs) are normally required [7, 116].

4 $${\text{O}}_{{2}}\, {\text{efficiency }}(\% ) = \frac{{2 \times \frac{{i_{{\text{R}}} }}{N}}}{{\left| {i_{{\text{D}}} } \right|\,\frac{{i_{{\text{R}}} }}{N}}} \times 100$$

where *i*_R_ is the ring current, *i*_D_ is the disk current, and *N* is the collection efficiency of the rotating ring disk electrode (RRDE), which is determined by the intrinsic parameters of the ring and disk electrodes. Calibration of *N* is required for accurate values [96].

In addition to the oxygen flow rate, temperature also affects the diffusion of O_2_. The ORR process is accelerated at high temperature due to enhanced diffusion of O_2_. However, the solubility of O_2_ is reduced at elevated temperatures, and this results in accelerated decomposition of H_2_O_2_ [20]. Thus, further studies are needed to investigate the complex effects of temperature on H_2_O_2_ electrosynthesis by the 2e^−^ ORR route.

### Electrodes

#### RRDE

RRDEs are widely used to quantify the performance of an electrocatalyst for 2e^−^–ORR production of H_2_O_2_ at the bench-top scale. H_2_O_2_ produced at the disk electrode is collected by the ring electrode and further oxidized back to O_2_. Both the disk and ring currents are recorded by an electrochemical workstation. Onset potentials and ring/disk currents can be used to assess the activity and selectivity of electrocatalysts in the ORR [117]. Specifically, a more positive onset potential indicates a lower energy barrier for the ORR, which results in better ORR electrocatalytic activity with lower energy consumption. By analyzing the currents of the ring and disk, the selectivity for H_2_O_2_ production can be quantified as the O_2_ efficiency (Eq. 4) or FE (*λ*_FE_%, Eq. 5) [7].

5 $${\text{FE }}\left( {\lambda_{{{\text{FE}}}} } \right) \, \% = \frac{{i_{{\text{R}}} }}{{N\left| {i_{{\text{D}}} } \right|}} \times 100$$

FE is used to evaluate the selectivity for H_2_O_2_ production in terms of the energy cost for H_2_O_2_ generation. In contrast, by calculating the number of electrons transferred with Eq. 6, the corresponding electrocatalytic selectivity can be determined. Thus, the percentage of 2e^−^ and 4e^−^ processes in the overall reaction are reflected directly. An *n* value of 2 corresponds to the 2e^−^ ORR route, while an *n* value of 4 indicates the 4e^−^ ORR pathway. Thus, an *n* value close to 2 indicates high selectivity for H_2_O_2_ production.

6 $${\text{Average number of transferred electrons}}:n = \frac{{4 \times \left| {i_{{\text{D}}} } \right|}}{{\left| {i_{{\text{D}}} } \right| + \frac{{i_{{\text{R}}} }}{N}}}$$

#### GDE

**Fig. 15:**
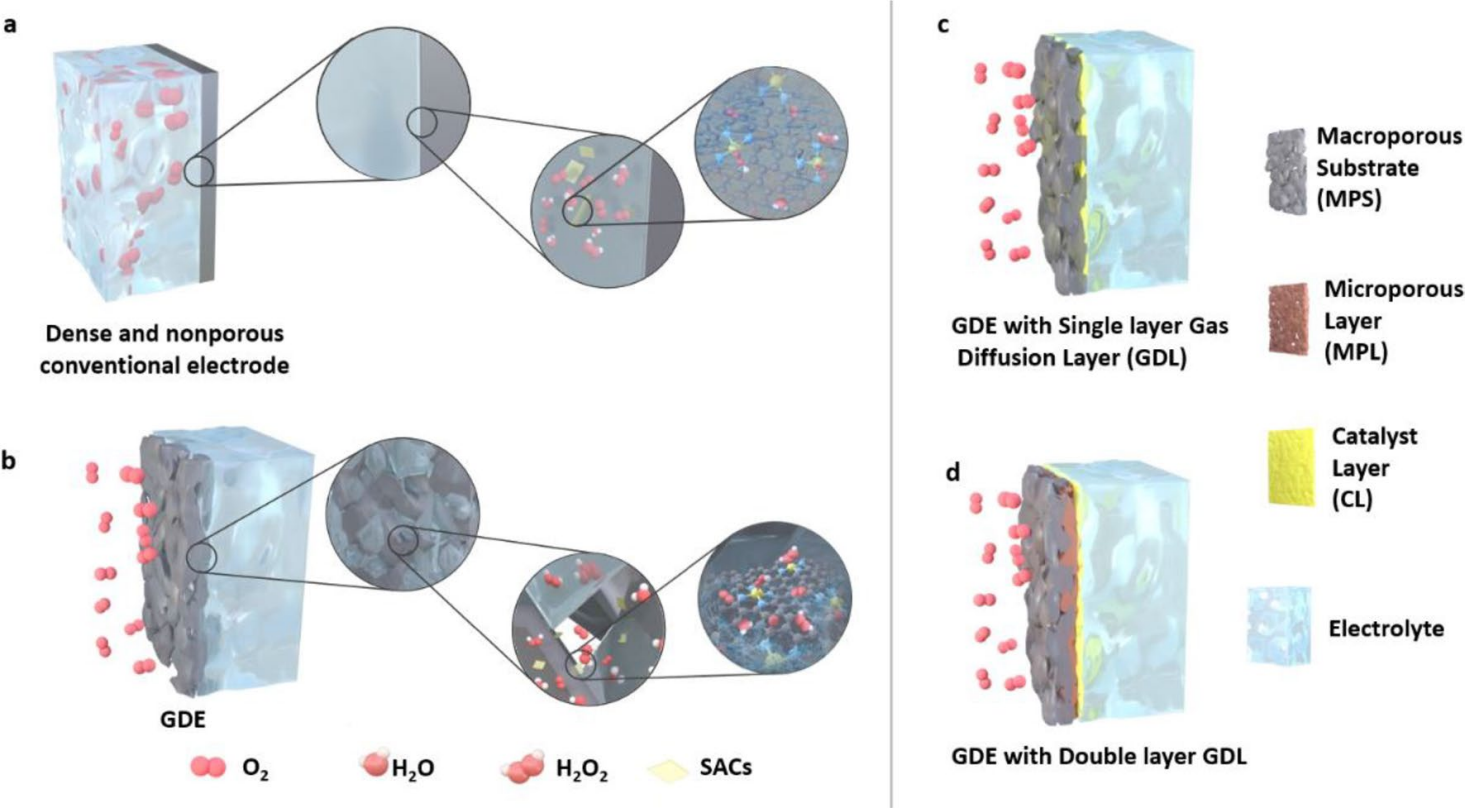
Schematic illustrations of **a** a dense and nonporous conventional electrode and **b** a gas diffusion electrode (GDE) used during the 2e^−^ ORR for H_2_O_2_ production with multiple spatial scales. **c** GDE with a single layer gas diffusion layer (GDL) and **d** GDE with a double layer GDL

It should be noted that an RRDE greatly facilitates the assessment of lab-scale electrocatalytic performance. However, in attempting to meet the criteria for practical applications, electrodes with larger catalyst loading areas and lower gas transport losses have drawn increasing attention. GDEs have been used in fuel cells and metal-air batteries for a long time and have recently been utilized in other electrochemical processes, including nitrogen reduction reactions and carbon dioxide reduction reactions [118–122]. Consequently, measurements of catalyst performance in larger-scale H_2_O_2_ production are usually performed with GDEs or with other types of conventional electrodes. Conventional electrodes (Fig. 15a) are usually immersed in electrolytes during electrochemical tests, and the low concentration of O_2_ in the electrolyte solution (~ 1.2 mM at 101.325 kPa, 0.1 M KOH) substantially limits the reaction efficiency [69]. In contrast, by employing a porous hydrophobic gas diffusion layer (GDL) as the interface between inflowing gas and the electrolyte, the diffusion and supply of oxygen for electrocatalysis on GDEs are much enhanced (Fig. 15b). During the reaction, O_2_ transferred through macropores and micropores in the GDL reacts with H^+^ (in acidic media) or H_2_O (in alkaline media) in the electrolyte at the catalyst layer (CL) to produce H_2_O_2_. In this case, a three-phase boundary is established, and a continuous supply of O_2_ to the catalyst is achieved, which largely overcomes the low solubility of O_2_ in the electrolyte solution. Thus, a GDE is typically constructed with a porous GDL and a catalyst layer (CL) deposited on the GDL, as is discussed in Sects. 4.3.2.1 and 4.3.2.2.

##### Gas Diffusion Layer (GDL)

A suitable GDL is required to conduct electrons effectively, it must be highly porous to support gas diffusion and highly hydrophobic to avoid flooding, and it must have a smooth and flat surface on which to load the CL. When excess electrolyte blocks the pores of the GDE, the gas diffusion pathways are either restricted or eliminated, and this ultimately leads to the total loss of GDE performance [123]. Thus, it is important to guarantee the presence of clear pathways for the GDL on a GDE. Generally, GDLs can be classified as one of two types, namely, single-layer GDLs (Fig. 15c) and double-layer GDLs (Fig. 15d).

Single-layer GDLs are macroporous substrates (MPSs) that are normally composed of carbon materials and hydrophobic agents, such as polytetrafluoroethylene (PTFE) and polyvinylidene fluoride (PVDF) [124, 125]. Carbon materials exhibit good conductivity and chemical stability, while hydrophobic agents impart great waterproof properties on single-layer GDLs. Additionally, metal-based (e.g., stainless steel, titanium) and PTFE-based (e.g., Gortex membrane) GDLs have also emerged as GDEs [126, 127].

**Fig. 16:**
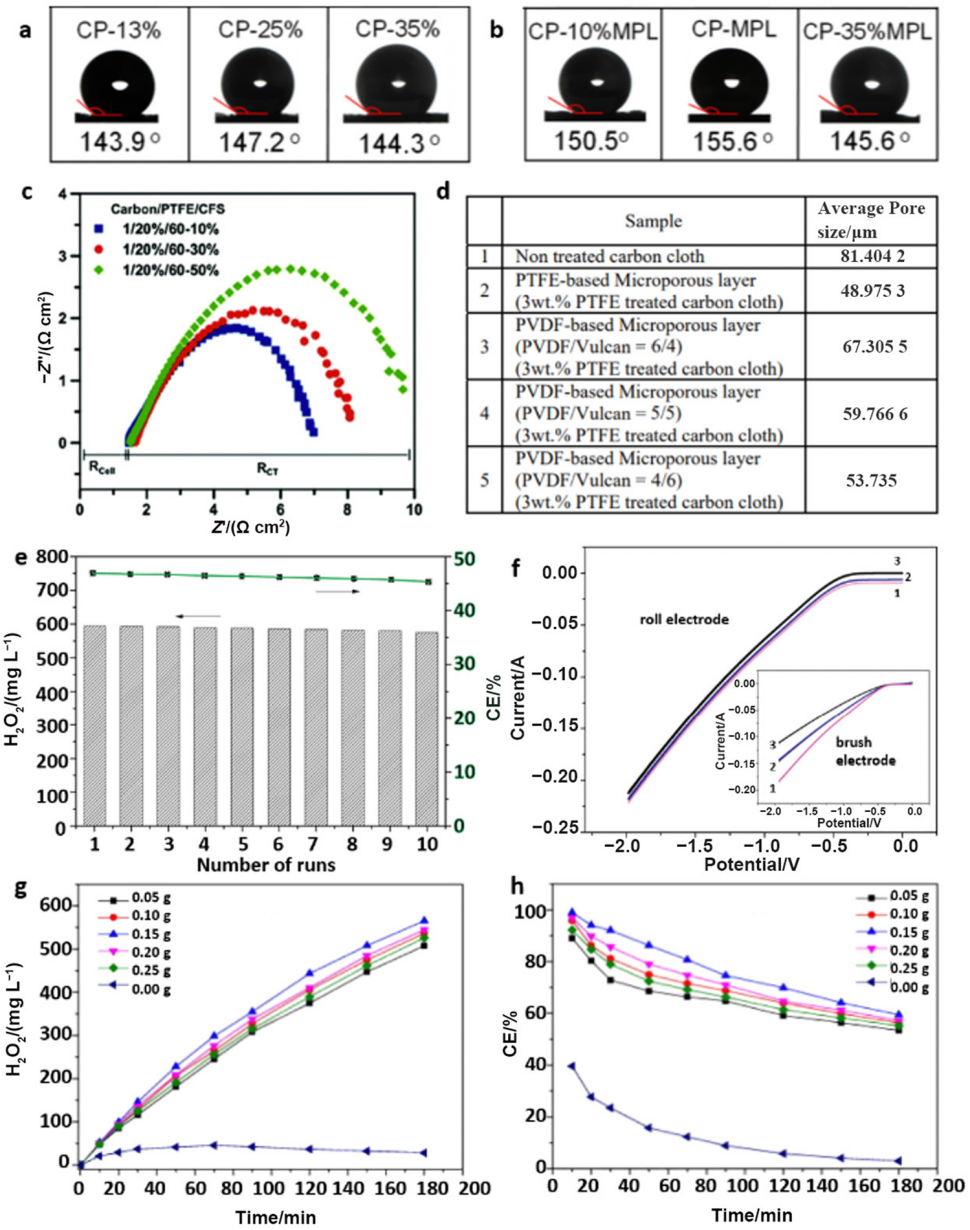
Contact angles of water droplets for Cu-loaded **a** single layer GDL (PTFE 13, 25, 35 wt.%, in carbon paper (CP)) and **b** dual-layer GDL (PTFE at 10, 25, 35 wt.% in a microporous layer (MPL), PTFE constant at 25 wt.% in CP) tested in 1 M KOH at 25 °C. Panels (**a, b)** are reprinted with permission from Ref. [128]. Copyright © 2020, John Wiley and Sons. **c** Effect of PTFE content in the macroporous substrate (10, 30, 50 wt.%) on electrical resistance in an Ag-loaded GDE. Reprinted with permission from Ref. [129]. Copyright © 2016, Elsevier. **d** Table of pore size distributions measured in a PTFE-based MPL and a PVDF-based MPL with different PVDF concentrations. Reprinted with permission from Ref. [125]. Copyright © 2009, IOP Science. **e** Stability test of rolling-made GDE in 10 continuous runs. in 0.05 M Na_2_SO_4_. **f** LSVs of the rolling-made GDE and brushing-made GDE in oxygen saturated 0.05 M Na_2_SO_4_. Panels (**e**, **f**) are reprinted with permission from Ref. [130]. Copyright © 2016, Elsevier. Effect of carbon black deposition amount on **g** the yields of H_2_O_2_ and **h** current efficiency (CE%). Panels (**g, h**) are reprinted with permission from Ref. [131]. Copyright © 2015, Elsevier

For single-layer GDLs, the type and content of hydrophobic agents serve as key factors determining hydrophobicity, gas permeability, and electric conductivity. The most commonly utilized hydrophobic agent is PTFE. Obviously, higher PTFE contents in GDLs result in higher hydrophobicities. Nonetheless, too much PTFE produces a rather coarse GDL surface, a higher electrical resistance, and undesired blockage of GDL pores, even though maximum hydrophobicity can be achieved, as shown in Fig. 16a, c [128, 129]. In addition to PTFE, hydrophobic PVDF is also used for fabrication of GDLs. PVDF is made with a lower melting temperature (~ 180 ℃) than PTFE (327 ℃), and PVDF exhibits lower costs, easier processing, and higher wear resistance [125]. The relationship between PVDF content and pore size in GDLs was investigated, as shown in Fig. 16d. With a decrease in PVDF content, pore sizes gradually decreased, thereby indicating lower surface resistance and higher gas permeability that are beneficial for GDLs. In addition to hydrophobic agents, the thickness of the GDL is another significant factor. Although more gaseous reactants can be transferred to the CL when the thickness is small, flooding is more likely to appear when the thickness is too small; thus, 190–250 nm is the most commonly adopted thickness for GDLs [129].

Dual-layer GDLs are composed of an MPS, as are single-layer GDLs, and a microporous layer (MPL) is sandwiched between the macroporous layer and the CL (Fig. 15d) for better interfacial electrical connection, water management, and flooding mitigation in cells [132, 133]. Although the MPL has a composition similar to that of single layer GDLs, the pore sizes and volumes of MPLs are much smaller than those observed at the macroporous level. Accordingly, some MPL nanoparticles fuse into the macropores of substrates to form an intermediate area rather than only attachment of the MPL to the MPS. The hydrophobic nature and nanoscale porosity enhance the oxygen transportation capability of the MPL while simultaneously blocking liquid penetration. As shown in Fig. 16a, b, compared to a single-layer GDL, a dual-layer GDL with the PTFE content in the MPL optimized (25% MPL) enhanced the hydrophobicity of the GDL. That is, an MPL can be utilized directly on single-layer GDLs to improve water repellence. With the introduction of an MPL, liquid and gas separation is maintained in the electrolyzer, electrode ohmic resistance is greatly reduced, contact resistance between the microporous layer and the CL is efficiently decreased, and the structural integrity of the electrolyzer is highly enhanced [128, 132, 133].

To limit the electrolyte to the CL side and offer a flat and smooth surface for the homogeneous coating of the CL, the MPL is required to exhibit optimal hydrophobicity. As illustrated in Fig. 14e, the extraordinary granular microporous structure of the MPL is particularly beneficial for diffusion of feed gases at the triple-phase boundary and two-phase interfaces to improve the corresponding reaction rate [128]. As with single-layer GDLs, the contents of hydrophobic agents, such as PTFE, in the MPL affect the electrical resistance and performance of electrocatalysts to some extent. Although superfluous addition of PTFE leads to an increase in electrical resistance, insufficient PTFE causes a loss of hydrophobicity (Fig. 16b), especially when the PTFE content drops below 20 wt.%, and flooding of the GDE occurs. For the CL coating, the thickness of the MPL should normally be well controlled within the range of 15–20 nm as a relatively smooth and flat substrate, and a thicker MPL offers an elongated gas diffusion pathway that may result in gas transfer restrictions [124, 129].

##### Catalyst Layer

As shown in Fig. 15c, d, CLs loaded with electrocatalytic materials are typically placed near the electrolyte side of the GDL. Generally, the conditions of the CL affect reaction pathways and even determine the efficiency of the desired 2e^−^ ORR process. To efficiently connect gas delivery and electrolyte flow channels, CLs must be provided with enough oxygen on the gas-feeding GDL side and sufficient electrolyte-catalyst contact on the other side, and this depends greatly on the parameters used for fabrication of the CL [134]. Typically, the porosity, fabrication method, and catalyst load directly affect the morphology, electrical conductivity, and ohmic resistance of the CL, which ultimately affects the performance of the GDE. In addition, the concentration of feed gas at the CL, the formation of triple-phase boundaries, and mass transport of reagents/products through the CL are also influenced by the surface properties of the CL [135, 136].

Obviously, the porosity of the CL influences the electrocatalytic performance of the GDE, since a highly porous structure facilitates the diffusion of reagents and products [137]. For instance, the relationship between pore architecture and the corresponding GDE performance for H_2_O_2_ electrogeneration via the 2e^−^ ORR was investigated [138]. After pretreatment with 20% nitric acid, the microporous surface area of graphite was increased by 24.51%, and this led to a 46.9% increase in the H_2_O_2_ yield rate. In addition to the intrinsic pore structures of catalysts, the content of ionic binder used during CL fabrication also affects porosity [139]. Generally, ionic binders create spanning networks with which to construct a large number of pores within the CL, connect catalysts, and transfer ions. Therefore, an appropriate ionic binder content is necessary. A low content results in attenuated ion transfer, increased aggregation of catalyst powder, and ultimately higher ohmic resistance, while an excess reduces the contact between the CL and the electrolyte; this leads to decreases in open pore volume and gas permeability and a subsequent increase in mass transport polarization, thus negatively influencing the ultimate performance of GDE. Moreover, higher usage of binder also results in a higher cost. Therefore, it is preferable to optimize the dosage of the binder by choosing appropriate methods for catalyst deposition.

Several methods, such as air-brushing, drop-casting, hand-painting, and rolling-made methods, are used for ink-based catalyst deposition. When loading the same catalyst, drop-casting and hand-painting CL usually would result in much smaller thickness than the well-defined and thicker CL made by air-brushing, which can be attributed to swift solvent evaporation and immediate fixation of ink mist sprays on the already solidified ink [140]. Although a thicker CL can result in enhanced resistance to diffusional mass transfer, a modified concentration of feed oxygen overcomes this. Moreover, the uneven CLs produced by drop-casting and hand-painting methods with aggregated catalysts contain fewer active electrocatalytic sites than CLs made by air-brushing [141].

Nonetheless, compared with the rolling-made method, air-brushing, drop-casting, and hand-painting methods give relatively coarser surfaces and are more labor-consuming for commercial applications. Novel rolling-made CLs that use carbon black as active materials were used as cathode electrodes for H_2_O_2_ electrogeneration [130]. The rolling-made CL provided both a high H_2_O_2_ production of 595 mg L^−1^ for 2 h and great stability with only 3.36% decay over at least 10 consecutive cycles, as shown in Fig. 16e. Compared with those of the brushing-made CL, the relatively more consistent LSV curves (Fig. 16f) of rolling-made CLs fully indicate the higher stability of the rolling-made CLs. Interestingly, this preparation method provides direct contact between the CL and the electrolyte without the utilization of ionic binder, which greatly reduces the cost. Although further breakthroughs in industrial-scale technology are still required, this binder-free method is worth pursuing.

In addition to the factors considered above, catalyst loading in the CL can also affect the mass transfer of reactants/products significantly, which leads to manipulation of the final reaction pathway. A higher loading amount offers more available catalytically active sites and allows higher current densities, but this hardly guarantees a better FE for the targeted product [142]. With alterations of the GDE potential at a certain current density, higher concentrations of intermediates may be produced, which would lead to changes in the product distribution of the 2e^−^ ORR. For example, various amounts of carbon black (0.05, 0.10, 0.15, 0.20 and 0.25 g) were investigated with CLs for GDEs used in the ORR [131]. Carbon black (0.15 g) provided the optimal electrocatalytic production of H_2_O_2_, 566 mg L^−1^ after 3 h at a current density of 7.1 mA cm^−2^, as well as the highest current efficiency (59.4%, CE%), as shown in Fig. 16 g, h; the use of other carbon black weights led lower yields of H_2_O_2_ due to alterations in intermediate concentrations and variations in gas permeability resulting from CL thickness.

## Design of Cells for H_2_O_2_-Producing Electrocatalysts

**Fig. 17:**
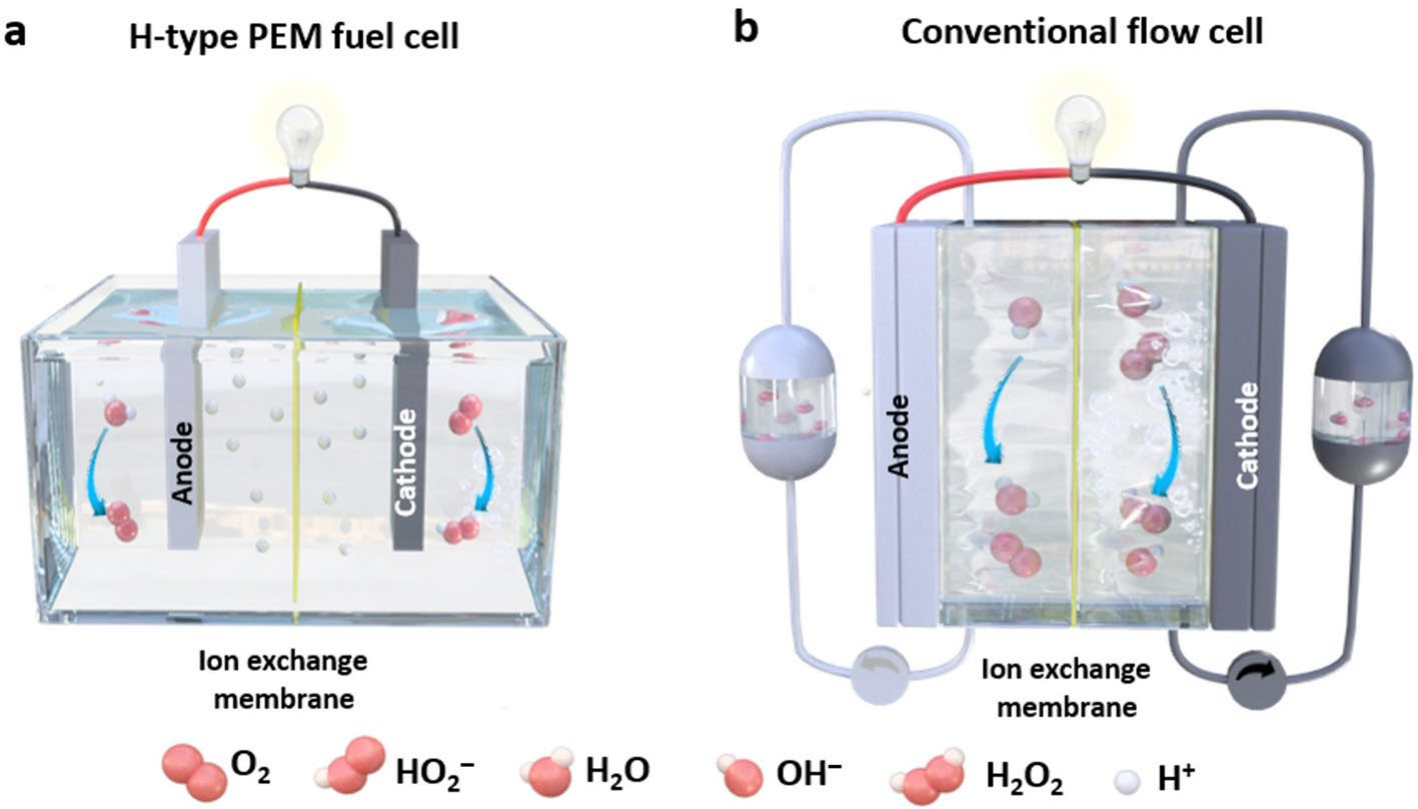
Schematic illustrations of various electrochemical 2e^−^ ORR processes for H_2_O_2_ production. **a** H-type PEM fuel cell and **b** conventional flow cell

To determine “real-scenario” performance accurately, catalysts must be tested in assembled cells, such as the electrolyzers shown in Fig. 17a, b. Yamanaka et al. conducted pioneering studies by combining electrochemical H_2_O_2_ production and H-type proton exchange membrane (PEM) fuel cells (Fig. 17a), in which protons were supplied by the evolution of O_2_ from water in the anode chamber [16]. This setup eliminated the use of H_2_, which eliminated the extra transportation costs and safety concerns arising from H_2_ usage. In 2018, Yamanaka and coworkers reported that the concentration of H_2_O_2_ produced in this configuration was as high as 18.7 wt.% when a Co–N_*x*_/C material was used as the cathode catalyst [143]. In addition to H-type fuel cells, conventional flow cells have also been used to produce H_2_O_2_ with O_2_ insufflation, and this was even combined with furfural oxidation to generate 2-furoic acid at the anode [6]. With this protocol, the levels of H_2_O_2_ generation at the cathode and 2-furoic acid generation at the anode reached 9.66 mol h^−1^ ${\mathrm{g}}_{\mathrm{cat}}^{-1}$ and 2.076 mol m^−2^ h^−1^, respectively, at a cell voltage of 1.8 V. Traditionally, the Ce(SO_4_)_2_ titration method is used to measure the concentration of H_2_O_2_ generated in the cell. In this process, the yellow color of the solution containing Ce^4+^ is dissipated by H_2_O_2_ reduction, which produces colorless Ce^3+^ (2Ce^4+^  + H_2_O_2_ → 2Ce^3+^  + 2H^+^  + O_2_) [96]. Ultraviolet–visible (UV) spectroscopy at a wavelength of approximately 316 nm was utilized to evaluate the concentration of Ce^4+^ before and after the reaction. Thus, the concentration of H_2_O_2_ produced (*c*(H_2_O_2_)) can be calculated from the amount of Ce^4+^ consumed (*c*(Ce^4+^)) with *c*(H_2_O_2_) = $\frac{1}{2}$  × *c*(Ce^4+^) [84]. Using the reaction time, the yield rate of H_2_O_2_ in the cell can be determined. In addition, the FE of cells (*λ*_FE cell_%) used for electrocatalytic H_2_O_2_ production via the 2e^−^ ORR and the corresponding energy efficiency (electricity-to-H_2_O_2_ efficiency, *λ*_EE_%) can also be quantified with Eqs. 7 and 8, respectively.

7 $${\text{FE }}\,{\text{of}}\,{\text{ cell }}\left( {\lambda_{{\text{FE cell}}} } \right)\% = \frac{2 \times n \times F}{C}$$

8 $${\text{and }}\,{\text{efficiency }}\left( {\lambda_{{{\text{EE}}}} } \right) \, \% \, = \frac{{(E_{{{\text{anode}}}}^{0} - E_{{{\text{cathode}}}}^{0} ) \times \lambda_{{\text{FE cell}}} }}{{V_{{{\text{cell}}}} }} \times 100$$

where, *n*—generated H_2_O_2_, mol; *F*—Faraday constant, 96 485 C mol^−1^; *C*—total amount of charge passed, C; ${E}_{\mathrm{anode}}^{0}$—theoretical potential of anodic reaction, V; ${E}_{\mathrm{cathode}}^{0}$—theoretical potential of cathodic reaction, V; ${V}_{\mathrm{cell}}$—uncompensated cell voltage, V.

Despite the continuous bubbling of the inlet oxygen gas that agitates the electrolytes in conventional cells (Fig. 17), the generated H_2_O_2_ still accumulates in the cathode region and exhibits a maximum concentration near the electrode. This not only impacts the dynamic environments of cells but also causes the conversion of H_2_O_2_ into H_2_O near the cathode, thereby reducing the apparent selectivity and total yield rate [144–146]. Thus, it is essential to develop advanced cell designs to minimize the gap between lab-scale and industrial-scale performance of the catalyst, which is very important in deploying lab-scale investigations into commercial devices for electrocatalysis.

Continuous flow cells, which steadily transport reactants and products toward and away from GDEs, are ideal configurations for overcoming the mass-transport and gas diffusion limitations of conventional cells [147]. Membrane-containing cells and microfluidic cells are two typical continuous flow cells. A single unit can be easily assembled in the laboratory, while enlarged stacks can be built by stacking multiple units; this effectively shortens the time for transition from research to practical applications [148]. However, there is little research on electrocatalytic production of H_2_O_2_ via the 2e^−^ ORR conducted in continuous flow cells due to the embryonic stage of development for the corresponding electrocatalysts.

**Fig. 18:**
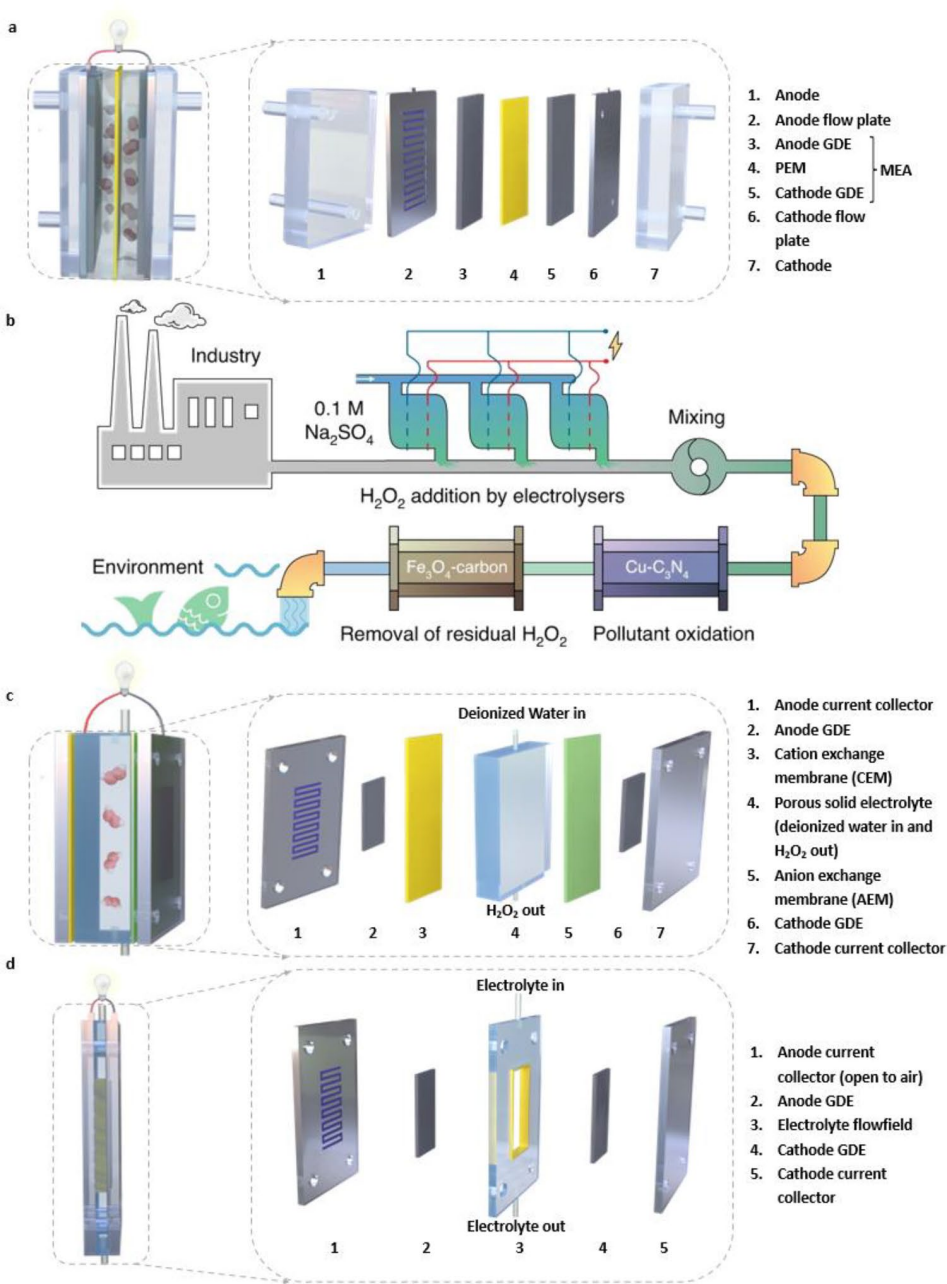
**a** Schematic illustration of a membrane-based flow cell containing a membrane electrode assembly. **b** Schematic drawing of a wastewater treatment system. Reprinted with permission from Ref. [152]. Copyright © 2020, Springer Nature. **c** Schematic illustration of a dual membrane flow cell for H_2_O_2_ synthesis on both the cathode and anode sides. **d** Schematic illustration of a nonmembrane microfluidic cell with a flow channel for liquid electrolytes to pass through

Membrane-based cells (Fig. 18a) are composed of a cathode and an anode separated by a polymer electrolyte membrane (PEM), which is capable of selective transmission of ions and suppression of product crossover. In such a cell, O_2_ is fed into the cathodic chamber for the ORR, while a water oxidation reaction (WOR, producing either H_2_O_2_ or O_2_) occurs at the anode. GDEs are utilized in the cell to improve the upper limits of cell performance [149]. A continuous flow of electrolyte solution past the cathode removes the generated H_2_O_2_ from the electrode surface; this prevents the aggregation of H_2_O_2_, which may be further reduced to H_2_O or decomposed. For instance, in such a membrane-containing continuous flow cell, a maximum H_2_O_2_ concentration of 1 400 ppm (1 ppm = 1 μmol mol^−1^) can be achieved in neutral conditions over long time periods when a conventional GDE is utilized [150]. Recently, another breakthrough was realized when a maximum steady-state H_2_O_2_ concentration of 3 000 ppm was produced at near-neutral pH, and this was achieved with a membrane electrode assembly (MEA) in a continuous flow cell [151]. To fabricate the MEA, hot pressing was conducted on the anode and the catalyst-coated GDE with sandwiched membrane at 135 °C and 7 584 kPa for 2.5 min (Fig. 18a), and this MEA was placed between the anode and cathode current collectors during testing. The MEA enabled direct feeding of gaseous O_2_ to the cathode rather than in the flowing catholyte, which greatly increased the oxygen concentration in the cathode region [149]. This also overcame the mass transport issues arising from gas diffusion and provided the opportunity to enhance electrocatalytic performance without elevating pressures or temperatures. Therefore, this “catholyte-free” flow cell shows promise for generating H_2_O_2_ at high concentrations [147].

Recently, a similar membrane-based cell with a gas tube connecting the tops of the cathode chamber and the oxygen feeding space was utilized to develop an efficient wastewater treatment system, which successfully solved two major challenges for current advanced oxidation processes (AOPs): simultaneous activation and sustainable production of H_2_O_2_ [152]. As shown in Fig. 18b, five steps were required for this wastewater treatment process. First, with the use of electricity and air, H_2_O_2_ was produced in a 0.1 M Na_2_SO_4_ electrolyte solution in an electrolyzer containing an electrocatalyst (e.g., Cu–C_3_N_4_, which continuously produces a pH-neutral 10 g L^−1^ H_2_O_2_ solution). Then, the resulting H_2_O_2_ solution was added to the untreated wastewater and mixed thoroughly. Next, the mixed liquids were passed through a Fenton filter, in which organic contaminants were oxidized by Cu–C_3_N_4_. After that, the liquid was continually passed through a Fe_3_O_4_-carbon filter, in which the remaining H_2_O_2_ was quenched (H_2_O_2_ removal efficiency > 99.9% for 100 h). Finally, the treated sewage with few toxic byproducts was discharged into the ecosystem. The promotion of this system indeed opens a new pathway for treatment of organic wastewater. However, the residual sulfates in the effluent, the high ratio of H_2_O_2_/total organic carbon required, and the need for scale-up to the industrial level will stimulate further development of the technology in the future.

In addition to the PEM-separated membrane-based cells introduced above, a novel membrane-based cell (Fig. 18c) that sandwiched a cation exchange membrane (CEM), a porous solid electrolyte (e.g., styrene–divinylbenzene copolymer microspheres functionalized with sulfonic acid groups), and layers of an anion exchange membrane (AEM) was reported to achieve the direct electrocatalytic production of pure H_2_O_2_ solutions [153]. The designs of the CEM and AEM efficiently prevented flooding of the GDE, which is typically caused by direct contact with liquid water. During the reaction, both anodic reactants (e.g., pure H_2_) and cathodic reactants (e.g., pure O_2_) were fed through the flow channels of the anode and cathode to the corresponding electrocatalysts, respectively. Moreover, the centrally located porous solid electrolyte accelerated the recombination of ions generated in both the anode (e.g., H^+^) and cathode (e.g., HO^2–^) chambers to produce pure H_2_O_2_. Compared to other systems, this design included a greatly reduced distance between the anode and cathode, resulting in an obvious decrease in ohmic losses. Finally, the generated H_2_O_2_ is dissolved and transferred out of the cell by the flowing deionized water in the middle layer. The resulting concentration of H_2_O_2_ can easily be manipulated up to 20 wt.% without further purification be controlling the rate for generation of HO^2–^ or the rate of deionized water flow.

However, the involvement of a membrane more or less increases the internal resistance of flow cells, which increases the consumption of electrical power to some extent. Consequently, an alternative configuration was proposed by Kenis in 2010, which was designed based on microfluidic cells without membranes (Fig. 18d) [154, 155]. In this configuration, the anode and cathode are separated by channel (volume ~ 0.15 mL) made with a thin sheet of poly(methyl methacrylate) allowing the liquid electrolyte to pass [155]. The feed O_2_ on the cathode side reacts at the interface of the electrolyte and the GDE to generate H_2_O_2_. Accurate control of the operating conditions for this kind of microfluidic cell has been shown to be a contributing factor in reaching high current densities for 2e^−^–ORR production of H_2_O_2_.

Recently, another microfluidic cell was designed to carry out electrocatalytic 2e^−^ ORR and 2e^−^ WOR processes on the cathode and anode sides, respectively, thus generating H_2_O_2_ at both electrodes, as shown in Fig. 18d [146]. This unique cell has a theoretical maximum *λ*_FE cell_% exceeding 100% for H_2_O_2_ production and enables high production efficiency because two H_2_O_2_ molecules can theoretically be produced in this microfluidic cell. An H_2_O_2_ production rate of 24 µmol min^−1^ and a maximum *λ*_FE cell_% of 153% were achieved in an electrolyte containing 1.0 M Na_2_CO_3_ and 4 mg mL^−1^ Na_2_SiO_3_. Ultimately, a solid adduct of Na_2_CO_3_ and H_2_O_2_ (Na_2_CO_3_·1.5H_2_O_2_) was directly extracted from the electrolyte after a continuous electrolysis process [5]. However, this adduct powder can only be stored stably for 2 months at most [147].

Although the risks and transportation and storage costs can be reduced for dilute H_2_O_2_ produced in such a decentralized way, the extra purification processes required to separate H_2_O_2_ and electrolyte impurities can be costly and tedious. To further eliminate the need for separation, a nonmembrane cell was developed to synthesize H_2_O_2_ via a quinone-mediated phase transfer process [156]. A high FE of above 95% was achieved with an H_2_O_2_ production rate of 0.12 mmol^−1^ cm^−2^ h^−1^ in this device. However, this process actually alternates between the one-step electrocatalytic 2e^−^–ORR process and multistep and phase-transfer catalytic processes. The additional cell impedance induced by phase transfer of the mediator and its reduction leads to new issues, which are beyond the scope of this review.

## Challenges and Opportunities

**Fig. 19:**
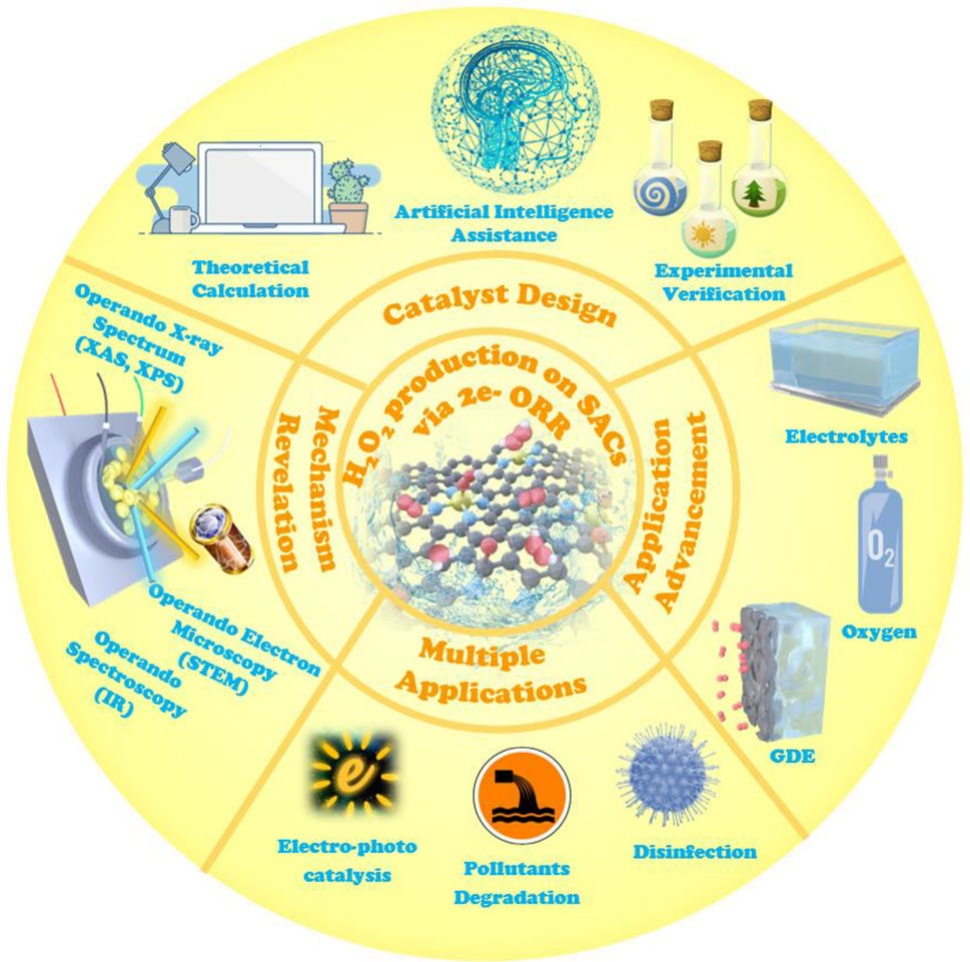
Schematic illustration of modification strategies for H_2_O_2_-producing SACs using the 2e^−^ ORR

Overall, a number of strategies for developing SACs exhibiting high electrocatalytic performance in the production of H_2_O_2_ via the 2e^−^–ORR route have been recently proposed and developed based on modifications of two features of SACs: electronic effects and geometric effects. These reported SACs possess the features of both heterogeneous and homogeneous catalysts, and they exhibit favorable electrocatalytic properties in acidic, neutral, and alkaline electrolyte solutions and well-defined structures. The development of cost-effective electrocatalysts exhibiting high activity, selectivity, and stability during H_2_O_2_ synthesis is one of the most significant challenges limiting the advancement of these state-of-the-art decentralized H_2_O_2_ production methods based on renewable energy sources. To support the performance-oriented design of new catalysts, theoretical predictions and in situ characterizations have been adopted to reveal some aspects of reaction mechanisms. However, the correlations between catalyst design and actual catalytic behavior still require further exploration. In the near future, in-depth investigations should be focused on the directions shown in Fig. 19.

### Catalyst Design: Theoretical Calculations Combined with Machine Learning

DFT calculations are widely used for calculations of adsorption energy used to screen catalysts and further elaborate their reaction mechanisms. However, compared with experimental studies, this approach seems oversimplified because it does not consider electrolyte effects (i.e., solvent, ion) and kinetic effects. Thus, deviations between theoretical predictions and experimental results may appear, and such distortions may also exist in the reaction mechanisms simulated theoretically.

In addition to more practical DFT calculation methodologies, additional attention should be focused on exploiting more efficient methods due to increases in complexity. Recently, artificial intelligence (AI), which uses a practical algorithm and software for data mining and analysis, has gradually emerged in the field of catalyst investigation [157]. Machine learning, a significant branch of AI, shows great potential for dealing with various problems in academic fields. With easy access to experimental data and rapid development of algorithms, machine learning and DFT calculations, catalyst design, model building, and studies of reaction mechanisms have been initiated for a variety of catalysts [76, 158–161]; included are catalysts for H_2_O_2_ production, ammonia decomposition, nitrogen fixation, ethanol reforming, and carbon dioxide reduction.

In addition to theoretical predictions, data mining of existing catalyst-related publications is another important direction for realizing the design of highly efficient catalysts and understanding reaction mechanisms with AI. For example, mining the details of synthetic routes to SACs, such as the selection of single metal atom centers and the design of their coordination environments, or the potential of catalysts for a specific reaction, such as H_2_O_2_ formation by the 2e^−^ ORR, will accelerate the discovery of optimal catalysts [162, 163]. Thus, the traditional time-consuming design of catalysts by DFT prediction and the corresponding experimental verification by trial-and-error will be updated with the implementation of a “big data” processing approach: machine learning. Further advances in our understanding of reaction mechanisms will also be achieved in a more efficient way with the help of machine learning. Thus, the evolution of parameters and algorithms for machine learning would facilitate significantly more targeted designs of SACs and more accurate investigations of reaction mechanisms, thereby turning an empirical approach into a mathematical process [164–167].

### Mechanism Revelation: Operando Investigations

During an electrocatalytic process, the structures of catalysts and active sites could change during long-term operation. Single-atom sites may aggregate or change their coordination environments during reactions. To further understand these phenomena, multiscale theoretical calculations must be accompanied closely by advanced experimental techniques. However, it is difficult to simultaneously capture and build atom- or molecular-level dynamic models with complex theoretical models of mass transport (i.e., reactants, products). Thanks to the evolution of reactors and operando investigations, operando XAS, X-ray photoelectron spectroscopy, STEM, Raman spectroscopy, and infrared spectroscopy are increasingly being used as experimental techniques tracking both the changes in catalysts and reaction processes. By doing so, mass transport processes, active site structures, intermediates and reaction pathways, mechanisms of catalyst degradation, and electrolyte effects can be monitored to probe the structure-performance correlations of catalysts for specific reactions [168]. Especially for SACs, the roles of isolated metal active centers and the coordination environments established during the reaction are the key to revealing structure-performance correlations, although the low metal loadings and isolated distribution continue to challenge the development of operando techniques. Thus, with the assistance of operando techniques, it is possible to study dynamic reaction processes continuously, and this will assist the development of comprehensive models for theoretical calculations and further aid in the design of highly efficient catalysts.

### Performance Enhancement: Material Assessment and Cell Optimization

Although the increased use of operando measurements has advanced cell design and new cell configurations have also been designed to overcome mass transfer problems during electrocatalytic reactions, the downstream separation of products remains a time-consuming and costly process. Instead of the widely used aqueous electrolytes, the development of ion-conducting polymer electrolytes, or even solid-state electrolytes, could constitute a rational approach to avoiding separation processes, and this has already been successfully exploited in cells for carbon dioxide reduction [169]. New electrolytes should simultaneously possess high conductivity, low cost, good resistance to reactant/product crossover, and long-term stability during reactions, which are the key challenges for development of electrolytes. In addition, more attention should be focused on the impact of oxygen feed on electrocatalytic H_2_O_2_ formation, including such factors as partial pressure, flow rate, and purity. GDEs, and especially the correlations between catalytic properties and the 3D pore configurations of GDEs, also require further investigation coupled with operando technologies and theoretical approaches to aid the development of cells.

### Multiple Applications: Cross-Disciplinary and Multifunctional Manipulation

In addition to electrocatalyzing H_2_O_2_ production via the 2e^−^ ORR process, our proposed design strategies for SAC fabrication can be further expanded into other related research fields, such as photocatalytic 2e^−^ ORR, electrocatalytic/photocatalytic 2e^−^ WOR, or even photoelectrochemical systems. For solar-driven H_2_O_2_ production, SACs can be deposited onto the surfaces of photoactive materials to serve as cocatalysts reducing reaction energy barriers, enhancing the efficiency of solar-to-H_2_O_2_ conversion and achieving high performance. Moreover, integrating a SAC-based cathode and a SAC-based anode to realize full H_2_O_2_ production seems to be more effective than using single-electrode systems, since electrons can be consumed more efficiently in this way. Subsequently, cross-disciplinary practical applications of catalytic H_2_O_2_ production systems, such as in organic pollutant degradation systems [152, 170] and water disinfection systems [66], would constitute a new direction in catalytic research and would also provide new pathways for the commercialization of academic catalytic processes.

In considering the future development of practical applications, scientists gradually noticed that SACs possessing only one type of active metallic site make it difficult to control adsorption of various reaction intermediates, thereby limiting the resulting catalytic efficiency [171, 172]. Recently, dual atomic catalysts (DACs) with two metallic active sites have emerged in various catalytic systems [173–175]. In future developments of SACs, more flexible active sites can be constructed [176]. Furthermore, due to the synergistic effects of different metal active sites, the center position of the d-band can be more effectively modified to optimize the interactions between active sites and reactants/intermediates and realize the use of multifunctionality. For example, when Fe–N–C/Zn–N–C active sites were introduced into the Co SAC/Cu SAC system, the selectivity for the 2e^−^ ORR was directly converted into selectivity for the 4e^−^ pathway [177, 178]. Although the development of DACs is still in its infancy, it can be foreseen that further design of DACs would become a new frontier in the development of SACs and provide new ideas for the fabrication of high-efficiency catalysts.

In this review, we proposed and summarized strategies for designing SACs and cells for the electrocatalytic production of H_2_O_2_ through the 2e^−^–ORR pathway, which is a promising and energy-efficient candidate to replace the current energy-intensive anthraquinone process in the future. We analyzed the electronic and geometric structures of SACs and discussed strategies for the modification of isolated metal atoms and neighboring coordination environments. Additionally, the correlations between SAC structure and electrocatalytic performance were illustrated, and the potential factors impacting the performance of SACs in H_2_O_2_ production were summarized. Finally, the challenges and opportunities for rational design of more targeted H_2_O_2_-producing SACs were highlighted. Overall, advances in the theoretical and experimental techniques used to advance electrocatalyst design and study the mechanisms of H_2_O_2_ production by the 2e^−^ ORR could also be instructive for other electrochemical conversion processes designed to replace centralized carbon-intensive approaches in the future.
